# Natural products targeting AMPK signaling pathway therapy, diabetes mellitus and its complications

**DOI:** 10.3389/fphar.2025.1534634

**Published:** 2025-02-03

**Authors:** Min Li, Lu Ding, Liyuan Cao, Zepeng Zhang, Xueyan Li, Zirui Li, Qinjing Xia, Kai Yin, Siyu Song, Zihan Wang, Haijian Du, Daqing Zhao, Xiangyan Li, Zeyu Wang

**Affiliations:** ^1^ Northeast Asia Research Institute of Traditional Chinese Medicine, Key Laboratory of Active Substances and Biological Mechanisms of Ginseng Efcacy, Ministry of Education, Jilin Provincial Key Laboratory of Bio-Macromolecules of Chinese Medicine, Changchun University of Chinese Medicine, Jilin, China; ^2^ Research Center of Traditional Chinese Medicine, The Affiliated Hospital to Changchun University of Chinese Medicine, Jilin, China

**Keywords:** AMPK, natural products, type 2 diabetes, complications, mechanism

## Abstract

Diabetes mellitus (DM) ranks among the most prevalent chronic metabolic diseases, characterized primarily by a persistent elevation in blood glucose levels. This condition typically stems from either insufficient insulin secretion or a functional defect in the insulin itself. Clinically, diabetes is primarily classified into type 1 diabetes mellitus (T1DM) and type 2 diabetes mellitus (T2DM), with T2DM comprising nearly 90% of all diagnosed cases. Notably, the global incidence of T2DM has surged dramatically over recent decades. The adenylate-activated protein kinase (AMPK) signaling pathway is crucial in regulating cellular energy metabolism, marking it as a significant therapeutic target for diabetes and related complications. Natural products, characterized by their diverse origins, multifaceted bioactivities, and relative safety, hold considerable promise in modulating the AMPK pathway. This review article explores the advances in research on natural products that target the AMPK signaling pathway, aiming to inform the development of innovative antidiabetic therapies.

## Introduction

Diabetes, along with its myriad complications, poses a significant global public health challenge, particularly with type 2 diabetes mellitus (T2DM), whose incidence is escalating markedly due to socio-economic development, lifestyle alterations, and an aging population (Ortiz-Martínez et al., 2022). The pathogenesis of diabetes is multifaceted, predominantly characterized by chronic hyperglycemia that is frequently associated with insulin resistance (IR) and compromised insulin secretion (Rabbani and Thornalley, 2024). Prolonged hyperglycemia can inflict damage on multiple organ systems, culminating in severe complications such as cardiovascular disease, kidney disease, and neuropathy (Li et al., 2024). Current clinically utilized drugs for managing diabetes mellitus and its complications are effective to a certain extent but frequently come with various side effects. Consequently, the development of novel, safe, and effective glucose-lowering therapies is of paramount importance.

In recent years, adenylate-activated protein kinase (AMPK), recognized as an intracellular energy sensor, has emerged as a pivotal regulator of glucose homeostasis, insulin sensitivity, and cellular functions (Cheng et al., 2023). An increasing body of research highlights the AMPK signaling pathway as a critical therapeutic target for diabetes and its complications. Activation of AMPK enhances glucose utilization and fatty acid oxidation, while simultaneously inhibiting gluconeogenesis and lipid synthesis. This multifaceted approach not only improves insulin resistance but also reduces blood glucose and lipid levels. Furthermore, AMPK plays a role in augmenting glucose transporter protein concentrations, facilitating glucose uptake into cells and thus lowering blood glucose levels (Behl et al., 2021). It also increases insulin sensitivity, diminishes insulin resistance, and curtails β-cell apoptosis, which collectively enhance insulin secretion. Additionally, AMPK activation mitigates oxidative stress, lipotoxicity, and inflammation, thereby alleviating diabetes-induced organ damage (Kakoti et al., 2024). These findings suggest that targeting the AMPK signaling pathway could offer novel strategies for managing diabetes and its associated complications.

Among natural products, numerous plant extracts have been identified as potential agents to ameliorate diabetes and its complications. These extracts exert their therapeutic effects by modulating the AMPK signaling pathway, thereby regulating glucose-lipid metabolism and improving insulin resistance, ultimately leading to reduced blood glucose levels (Rashid et al., 2022). For instance, treatment with hawthorn acid has been shown to mitigate body weight loss, blood glucose levels, and renal structural and functional damage in diabetic mice (Gao and Wu, 2022). Notably, hawthorn acid activates the renal AMPK/SIRT1 signaling pathway, significantly reducing oxidative stress and inflammation in the kidneys of diabetic rats. These findings underscore the potential of natural products as novel drug sources for diabetes treatment. This paper explores the pathogenesis of diabetes, risk factors, and the mechanisms of the AMPK signaling pathway, summarizing the interconnections among them. It primarily highlights the advancements in research on natural AMPK activators, reinforcing the role of natural products in diabetes prevention and treatment.

## Search strategy and selection criteria

### Search strategy

This review conducted literature searches across several databases including Scopus, PubMed, Web of Science, SpringerLink, and Science Direct, employing a combination of specific keywords and related terms. Key terms used in the search were “AMPK,” “natural product,” and “diabetes,” which were further combined with “type 2 diabetes,” “hyperglycemia,” “cardiovascular disease,” “nephropathy,” “neuropathy,” “complications,” “mechanism,” “ phenolic metabolites,” “terpenoids,” “Terpenoids,” “Quinones,” and “alkaloids.” The final search was completed in September 2024, and the searches were limited to literature published in English.

### Selection criteria

References were screened based on their relevance to the topic. Initially, titles of high-value references were read to assess their pertinence. This was followed by a title-specific search, and ultimately, a thorough reading of the entire articles. References and duplicate studies that did not align with the review’s focus were excluded. Inclusion criteria were set as follows: studies must center on AMPK, diabetes and its complications, or natural products; the research must be related to diabetes; and the studies should explore AMPK mechanisms. Exclusion criteria included: unclear research subjects, methodologies, or mechanisms of action; unreliable or low-quality publications; and duplicate publications and studies. To ensure the reliability of the selected studies, three researchers (ML, LD, and LC) independently screened and analyzed all the literature during the review process.

## Overview of diabetes mellitus

### Epidemiological characteristics of diabetes

Diabetes mellitus is characterized by metabolic disorders stemming from various factors, with chronic hyperglycemia being a primary symptom. Normally, the body can metabolize sugar to maintain a stable internal environment. However, diabetic patients often exhibit insufficient insulin secretion and impaired functionality, leading to metabolic imbalances in carbohydrates, fats, and proteins. This can result in chronic damage and functional disabilities across multiple organs (Templer et al., 2024).Consequently, managing blood sugar levels can mitigate cardiovascular diseases to an extent. Moreover, the epidemiology of diabetes exhibits regional variations (Abudureyimu et al., 2022). In some developing countries, the prevalence of diabetes is rising more rapidly due to economic development, lifestyle changes, and uneven distribution of medical resources (Chan et al., 2009). For example, according to the International Diabetes Federation (IDF) report, the number of diabetes patients in China have been exceed 140 million currently (Sun et al., 2022). Additionally, variations in genetic background, dietary habits, and physical activity levels among different populations contribute to regional disparities in diabetes risk (Shah et al., 2022).

### Pathogenesis

Over 90% of diabetes diagnoses are T2DM, a chronic metabolic disorder primarily characterized by a relative lack of insulin. This insufficiency is often due to factors such as reduced secretion, development of IR in visceral tissues, and loss of compensatory mechanisms, leading to persistently high blood glucose levels (Liu et al., 2024). Such conditions may precipitate metabolic syndrome and trigger complications, notably chronic inflammation (Kato et al., 2020). The onset of T2DM is heavily influenced by lifestyle factors, including being overweight, insufficient physical activity, poor dietary habits, daily stress, and genetic predispositions (Silva et al., 2024). According to the latest findings presented at an International Diabetes Federation online conference, inflammatory responses and oxidative stress are critical in the progression of T2DM (Ogurtsova et al., 2022). Prolonged hyperglycemia stimulates the production of reactive oxygen species, triggering oxidative stress and damaging proteins, lipids, and DNA within cells (Clemen et al., 2024). Concurrently, elevated levels of inflammatory markers like tumor necrosis factor-α (TNF-α) and interleukin-6 (IL-6) in individuals with T2DM exacerbate IR and impair beta-cell function in the pancreas (Mukherjee et al., 2021; Park et al., 2021; Fagarasan et al., 2023; Habibi et al., 2024). Furthermore, the dysregulation of intestinal flora is intricately linked to T2DM development (Martel et al., 2022; Yang et al., 2023). Poor dietary and lifestyle choices can alter the gut microbiota, reducing beneficial bacteria and increasing harmful ones. These shifts can disrupt nutrient absorption and metabolism, interfere with insulin signaling, and enhance inflammatory responses, thus heightening T2DM risk (Martel et al., 2022).

## Overview of AMPK signaling pathways

Adenylate-activated protein kinase (AMPK), also known as AMP-dependent protein kinase, is a pivotal element in diabetes research and other metabolism-related disorders. It participates extensively in various physiological processes, including cellular signaling, and affects a multitude of systems and tissue functions throughout the body (Herzig and Shaw, 2018; Geng et al., 2021). The AMPK signaling pathway also interacts with other critical metabolic regulators, collaborating with members of the peroxisome proliferator-activated receptor (PPAR) family to jointly regulate fat metabolism and insulin sensitivity (AL-Ishaq et al., 2019; Gong et al., 2024; Shi et al., 2024).

### AMPK acts on the human body

AMPK acts in several parts of the body, as described below: AMPK regulates mitochondrial biogenesis and function, thus enhancing cellular energy production capacities (Herzig and Shaw, 2018). In muscle tissue, AMPK activation boosts the expression of glucose transporters, facilitating glucose uptake and utilization, which in turn aids in blood glucose control (Kido et al., 2023). In the liver, it suppresses fat synthesis and gluconeogenesis while promoting fatty acid oxidation, thereby helping to maintain the liver’s energy balance (Deja et al., 2024). In adipose tissue, AMPK regulates both lipolysis and synthesis, influencing the metabolic activities of fat cells (De Melo et al., 2024). In the brain, particularly in the hypothalamic region, AMPK is involved in the regulation of appetite. In addition, AMPK protects neurons from oxidative stress, ischaemia and other injuries, which is potentially significant for maintaining normal brain function and preventing neurodegenerative diseases (Huo et al., 2023). In cardiac tissue, AMPK has an important regulatory role in the energy metabolism of cardiomyocytes (Persad and Lopaschuk, 2022). Overall, the AMPK signaling pathway is essential for maintaining metabolic balance across various biological aspects. Advancing research into the AMPK signaling pathway promises to uncover novel insights and strategies for treating diabetes mellitus and other metabolic diseases.

### Activation of the AMPK signaling pathway

The activation of AMPK is a complex process that primarily occurs through the following pathways: when cellular energy levels decrease, the AMP/ATP ratio increases, causing AMP to bind directly to the γ-subunit of AMPK, which induces a conformational change and subsequently activates its catalytic activity. This direct AMP binding is a fast and effective way of AMPK activation (Shu et al., 2023). Secondly, upstream kinases such as liver kinase B1 (LKB1) are also activated under energetic stress and activate AMPK by phosphorylating the threonine 172 site on the α-subunit of AMPK. This process requires the formation of a complex between LKB1 and AMPK and a phosphorylation reaction under specific conditions (Qiu et al., 2023). In addition, calcium ion/calmodulin-dependent protein kinase kinase β (CAMKKβ) is another important upstream kinase of AMPK. Under conditions of elevated calcium ion concentration, CAMKKβ is able to directly phosphorylate the α subunit of AMPK, thereby activating AMPK (Tokumitsu and Sakagami, 2022). In addition to the above pathways, the activation of AMPK may also be regulated by other factors, such as hormones, nutrients, and environmental factors. These factors can regulate the activity of AMPK by affecting its upstream signaling pathway or by directly interacting with AMPK. Furthermore, α-subunit phosphatase regulates this pathway by impacting the expression of specific proteins on cell membranes (Ashrafizadeh et al., 2021). The modulation of AMPK signaling involves complex interactions with other intracellular pathways, notably with the insulin signaling pathway, which is critical in energy metabolism regulation. Active insulin signaling can suppress AMPK activity to some extent, promoting anabolic processes; conversely, in states of energy deficiency, AMPK is activated, providing feedback to the insulin signaling pathway to maintain cellular energy equilibrium. Additionally, AMPK signaling is intricately linked to the autophagy signaling pathway.

**FIGURE 1:**
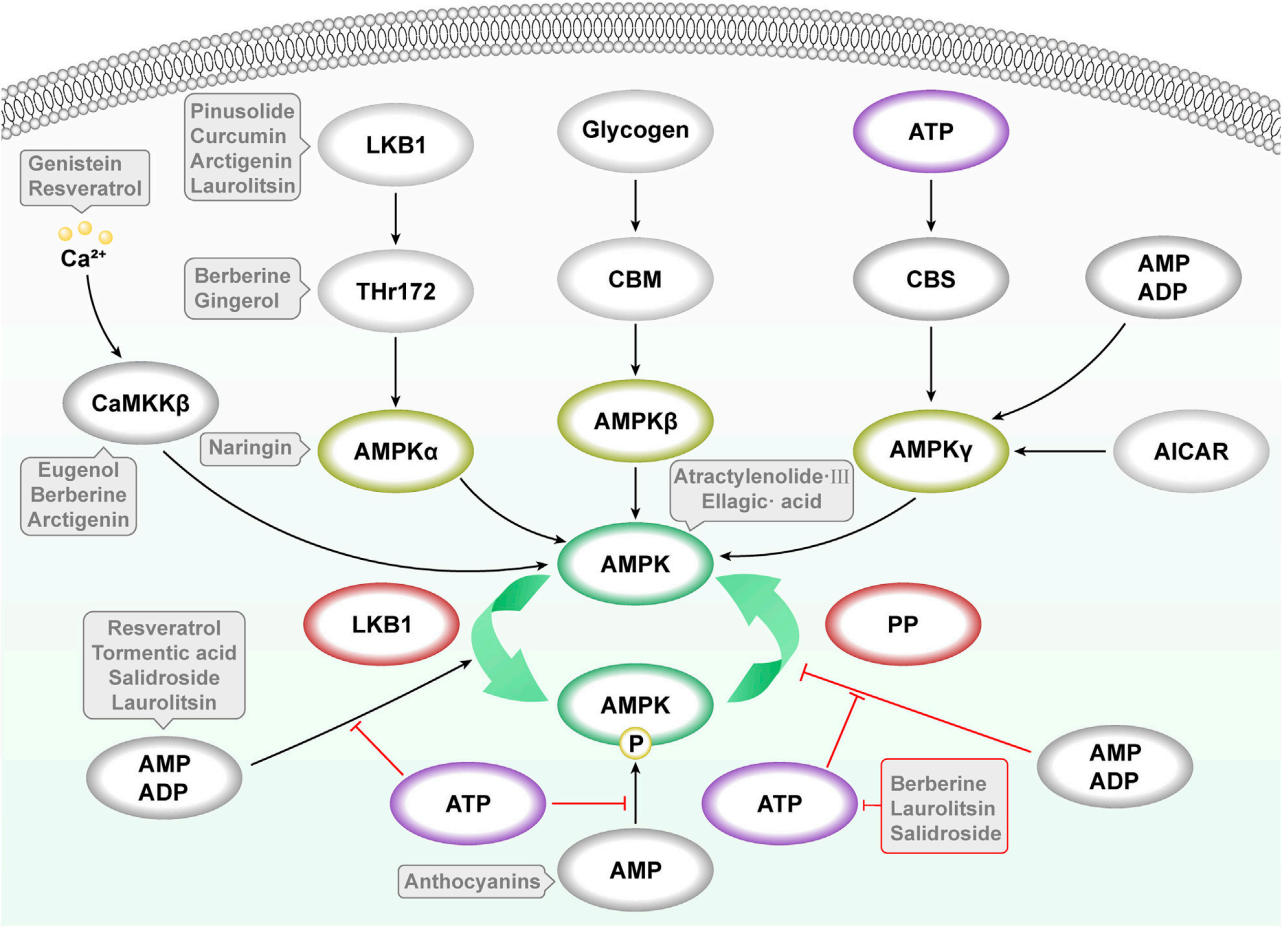
Canonical pathways for AMPK activation by adenine nucleotides and Ca^2+^-dependent mechanism mediated by CaMKKβ. 1) Activation of AMPK by promotion of Thr172 phosphorylation; 2) allosteric activation by AMP binding; 3) inhibition of Thr172 dephosphorylation by PP. CaMKKβ = calcium/calmodulin-dependent kinase kinase β; LKB1 = liver kinase B1, PP = protein phosphatases. Notations: Black Arrow (↓): Indicates promotion. Red Rough Arrow (⊥): Indicates inhibition.

AMPK is composed of three subunits: α (catalytic subunit), β (regulatory subunit), and γ (regulatory subunit). The activation of AMPK depends on several factors, primarily energy status: as cellular ATP levels decrease, AMP (adenosine monophosphate) and ADP (adenosine diphosphate) levels increase. These molecules bind to the γ subunit, promoting the activation of AMPK (Francini et al., 2019) (Figure 1).

Kinase phosphorylation also plays a crucial role; threshold activation of AMPK typically requires further phosphorylation, often mediated by kinases such as LKB1 (Ai et al., 2023), CaMKKβ (Hawley et al., 2005; Woods et al., 2005), etc. Once activated, AMPK influences a range of downstream target proteins to alter the metabolic state of the cell, primarily by enhancing glucose uptake and oxidation, promoting fatty acid oxidation, and stimulating mitochondrial biogenesis (Herzig and Shaw, 2018) (Figure 1).

AMPK activation is a complex, multistep process involving the regulation of multiple signaling pathways, enabling it to play a key metabolic role in diverse physiological and pathological conditions.

## Relationship between AMPK signaling pathway and DM

**FIGURE 2:**
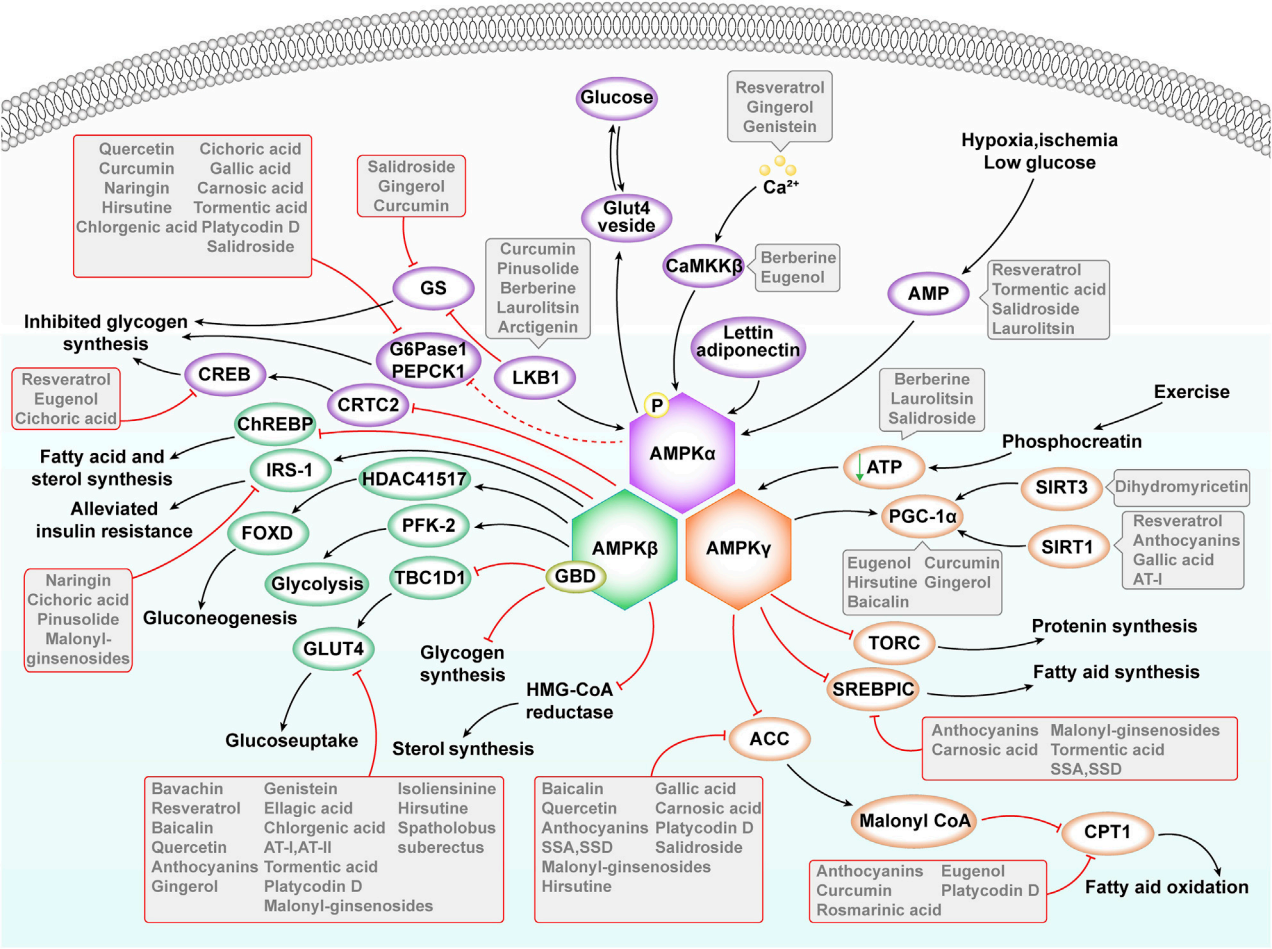
Main AMPK activators and downstream targets that regulate lipid, carbohydrate and protein metabolism. ACC, Acetil Coenzyme A Carboxylase; CaMKKβ, Calcium/Calmodulin-Dependent Kinase Β; CPT1, Carnitine Palmitoyltransferase 1; GBD, Glycogen-Binding Domain; GLUT 4: Glucose Transporters 4; HMG-CoA reductase, 3-hydroxy-3-methyl-glutaryl-coenzyme A reductase; LKB1, Liver Kinase B1; PFK-2, 6-Phosphofructo-2-Kinase 2; SREBP-1c, Sterol Response Element Binding Protein-1c; TORC, Target of Rapamycin Complex. GLUT 2: Glucose Transporters 2; Notations: Black Arrow (↓): Indicates promotion. Red Rough Arrow (⊥): Indicates inhibition.

After entering the body, γ-ketoate dehydrogenase catalyzes the conversion of glucose to lactic acid, which is subsequently transformed into carbon dioxide and excreted through the blood vessel walls (Cui et al., 2022). In the liver, glucose transport is facilitated by GLUT2 (Glucose Transporters 2), which is independent of IR involvement (Thorens, 2015). In patients with T2DM, persistently high blood glucose levels lead to an unusually high uptake of glucose by the liver. This uptake simultaneously inhibits the activity of glycogen synthase kinase 3β (GSK3β), thereby initiating the process of glycogen synthesis (Peng et al., 2024) (Figure 2).

### AMPK is associated with apoptosis, inflammation and oxidative stress

AMPK signaling emerges as a promising therapeutic target for various diseases. In the context of inflammatory bowel disease, dagliflozin has been observed to diminish mammalian target of rapamycin (mTOR) levels by enhancing AMPK phosphorylation (Arab et al., 2021). This, in turn, initiates autophagic mechanisms and decelerates apoptosis. Similarly, in treating non-alcoholic fatty liver disease (NAFLD), kynurenine boosts autophagy by elevating AMPK levels, effectively mitigating symptoms associated with NAFLD (Liu et al., 2020). Additionally, reducing caspase-6 aspartic acid concentrations can significantly enhance AMPK levels, inhibiting the progression of NAFLD cell death (Liang et al., 2021). The AMPK signaling pathway is also intricately linked with inflammation related to disease. Amyloid beta (Aβ) can provoke an inflammatory response by increasing the concentration of NLRP3 (NOD-like receptor thermal protein domain associated protein 3) (Qiu et al., 2020). In contrast, lychee seed polyphenols can activate the AMPK-autophagy axis, potentially ameliorating inflammatory conditions. Both lychee seed polyphenols and doxorubicin exhibit significant anti-inflammatory effects in acute pancreatitis. While doxorubicin is known to cause liver injury leading to necrosis and fibrosis in rats, lychee seed extract may alleviate these pathological changes in liver tissue. These findings confirm the critical role of cell-cell interactions in inflammatory liver diseases, although the interrelationships between these mechanisms remain to be fully elucidated. Research indicates that increasing AMPK levels can effectively reduce mTOR and activate Serine/threonine unc-51-like kinase 1 (ULK1), entering the initiation phase of autophagy. This process helps in preventing the formation of NLRP3 inflammatory vesicles (Xiong et al., 2021). Moreover, activating the AMPK signal can effectively suppress the production of inflammatory factors, thus slowing the onset and progression of inflammation (Liu et al., 2020) (Figure 2).

Additionally, ongoing research continues to explore the relationship between AMPK and key cellular processes such as apoptosis, inflammation, and oxidative stress. Recent studies have identified certain bioactive peptides that modulate AMPK activity, further influencing these pathological cellular processes. Moreover, exercise, as a non-pharmacological intervention, has been demonstrated to activate the AMPK signaling pathway, reducing inflammation, oxidative stress, and the risk of apoptosis (Saha et al., 2014). In the context of disease prevention and management, integrating lifestyle modifications, dietary optimizations, and appropriate exercise, along with the development of targeted AMPK-modulating drugs, could offer new avenues for combating diseases related to apoptosis, inflammation, and oxidative stress (Hu et al., 2001). Furthermore, the advancement of gene editing technologies holds the potential for precise manipulation of AMPK-related gene expression in the future, enabling more effective interventions in these pathological processes. In conclusion, the continued investigation into AMPK’s role in apoptosis, inflammation, and oxidative stress is poised to yield significant breakthroughs and innovations in medical science (Figure 2).

### AMPK, glucose uptake, and metabolism

Research into AMPK’s role in glucose uptake and metabolism continues to deepen. Recent studies have identified specific environmental factors, such as hypoxia (Mungai et al., 2011; Sallé-Lefort et al., 2016) and hypothermia (Chen D.-Q et al., 2022), that may influence glucose uptake and metabolism through their effects on AMPK signaling pathways. For instance, hypoxic conditions can stimulate cells to activate AMPK, thereby enhancing glucose uptake and utilization to satisfy energy requirements. Additionally, certain natural products may exert a synergistic effect on the regulation of glucose uptake and metabolism by AMPK. Combinations of specific plant extracts with particular nutrients might more effectively activate AMPK signaling, thereby improving glucose transport and metabolic efficiency. In clinical applications, the development of new therapeutic strategies based on AMPK’s role in regulating glucose uptake and metabolism holds considerable promise. Coupling these strategies with gene therapy technologies to precisely regulate AMPK-related gene expression, or developing novel AMPK activators, could provide innovative treatments for diabetes and other metabolic diseases. In conclusion, ongoing research on AMPK and its impact on glucose uptake and metabolism continues to generate fresh insights and methodologies for enhancing the treatment and prevention of metabolic disorders (Figure 2).

## Advances in research on AMPK activators in natural products

### Medicinal plants as potential sources of active metabolites

AMPK activators are categorized into two main types: direct and indirect activators. Indirect activators function by promoting the accumulation of AMP or calcium, thereby stimulating AMPK activity indirectly through changes in cellular ATP, ADP, or AMP levels. Direct activators, on the other hand, bind directly to AMPK and activate it without the influence of these nucleotides (Tamura et al., 2022). In addition to pharmaceuticals, a wide array of phytochemicals, including over 100 natural products or plant extracts such as polyphenols, have been identified as AMPK activators (Francini et al., 2019). However, the precise mechanisms of action for many of these metabolites remain elusive. Notable natural products thought to activate AMPK include phenolic metabolites like resveratrol, alkaloids such as berberine, and salicylate (Villarroel-Vicente et al., 2021). These anti-diabetic plants are seen as promising treatments for metabolic syndrome, with extensive research dedicated to exploring their potential. Many studies have identified promising candidate plants that might yield novel drug metabolites. For instance, recent research summarized in Table 1 focuses on phenolic metabolites in natural products as potential anti-diabetic drugs that activate AMPK. The variety of potential sources for active metabolites in medicinal plants is vast and exceeds those currently identified. As research progresses, an increasing number of secondary metabolites from various plant species are being discovered that have the potential to activate AMPK. Specific terpenoids and flavonoid derivatives, for example, may represent new sources of AMPK activators. Furthermore, broader screening and research into medicinal plants across different regions and ecological environments are likely to uncover more metabolites with unique activities. The utilization of modern biotechnology, including genetic engineering and synthetic biology, can enhance the efficacy and specificity of these active metabolites in activating AMPK. Additionally, through multidisciplinary collaboration involving experts in botany, chemistry, biology, and medicine, the mechanisms and potential applications of active metabolites in medicinal plants can be more thoroughly explored, paving the way for the development of new anti-diabetic drugs. Moreover, modern biotechnology, including genetic engineering and synthetic biology, can be employed to optimize and enhance the active metabolites in medicinal plants, improving their efficiency and specificity in activating AMPK. Additionally, through multidisciplinary cooperation and the combined efforts of experts in botany, chemistry, biology, medicine, and other fields, the mechanisms of action and potential application values of active metabolites in medicinal plants can be more thoroughly explored. This approach could significantly expand the possibilities for developing new anti-diabetic drugs.

**TABLE 1:** Natural products activate AMPK for the management of diabetes mellitus.

Natural components	Classification	Molecular formula	Chemical structure	Source	Model and dose		Mechanisms	Specific effects	Refs.
Eugenol	Phenols	C_10_H_12_O_2_	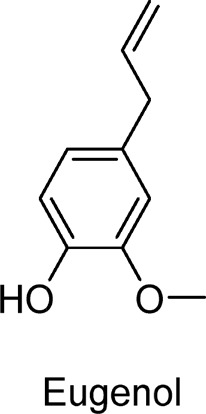	*Eugenia caryophyllata* Thunb	HepG2 cells, primary rat hepatocytes, high-fat diet (HFD)-fed C57BL/6J mice	20–40 mg/kg (mice), 100 μM (*in vitro* for glucose production assays)	AMPK activation via CAMKK, suppression of CREB-CRTC2 complex, inhibition of gluconeogenic genes (PEPCK, G6Pase)	Decreases hepatic glucose production, lowers blood glucose and insulin levels, improves glucose tolerance, inhibits gluconeogenesis, and activates AMPK signaling, potentially beneficial for managing type 2 diabetes.	Jeong et al. (2014)
[6]-Gingerol	Phenols	C_17_H_26_O_4_	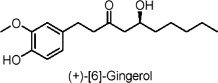	*Zingiber officinale* (ginger root)	L6 myotubes, RIN-5F pancreatic β-cells, db/db diabetic mice	50–100 µM (*in vitro*), 0.05% dietary supplementation (*in vivo* for 4 weeks)	AMPK activation, GLUT4 translocation, reduced ROS in β-cells, suppression of gluconeogenesis via G6Pase and PEPCK	Enhances glucose uptake, protects pancreatic β-cells from oxidative stress, lowers fasting blood glucose, improves glucose tolerance, reduces lipid peroxidation and serum TNF-α levels, regulates hepatic glucose metabolism.	Jin et al. (2015)
					High-fat high-carbohydrate (HFHC) diet-fed rats, L6 skeletal muscle cells	100–200 mg/kg (*in vivo*), 50–150 µM (*in vitro*)	AMPK activation, increased PGC-1α expression, enhanced mitochondrial biogenesis	Prevents insulin resistance, reduces fasting glucose and insulin levels, improves glucose tolerance, enhances energy metabolism, reduces lipid peroxidation, and increases mitochondrial content.	Li et al. (2014)
					L6 myotubes (skeletal muscle cells), HepG2 hepatocytes	50–150 µM (*in vitro*)	AMPK activation, GLUT4 translocation, intracellular Ca^2^⁺ increase via CaMKK2, increased PGC-1α expression	Enhances glucose uptake, stimulates mitochondrial biogenesis, improves insulin sensitivity, regulates glucose metabolism through AMPK signaling, and reduces insulin resistance.	Li et al. (2013)
					High-fat diet-induced insulin resistance in rats, L6 myotubes, HuH-7 hepatocytes	100–200 mg/kg (*in vivo*), 50–150 µM (*in vitro*)	AMPK activation, GLUT4 translocation, PGC-1α upregulation, NF-κB inhibition, COX-2 suppression	Improves glucose uptake, reduces insulin resistance, enhances mitochondrial biogenesis, inhibits hepatic inflammation, reduces oxidative stress, lowers fasting glucose and lipid levels.	Roufogalis (2014)
Chlorogenic acid	Polyphenols	C_16_H_18_O_9_	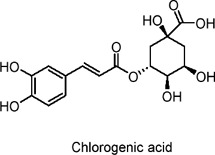	Coffee, various fruits	Leprdb/db mice (T2DM model), HepG2 hepatocytes	250 mg/kg/day (mice), 2 mM for 24 h (HepG2 cells)	AMPK activation, downregulation of G6Pase, increase in GLUT4	Lowers blood glucose, reduces gluconeogenesis, enhances glucose uptake in skeletal muscle, inhibits fatty acid synthesis, improves lipid profile, increases insulin sensitivity, reduces body weight and hepatic lipid accumulation	Ong et al. (2013)
Cichoric acid	Polyphenols	C_16_H_18_O_8_	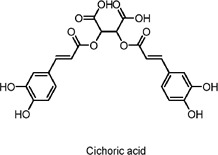	*Echinacea purpurea*, Chicory root, other edible plants	Streptozotocin-induced diabetic C57BL/6J mice, Glucosamine-induced HepG2 cells	60 mg/kg/day (mice), 100 μM (HepG2 cells)	AMPK pathway activation, PI3K/AKT pathway, Nrf2-Keap1 activation	Inhibits hepatic injury, activates antioxidant enzymes, reduces gluconeogenesis, increases glycogen synthesis, improves insulin resistance, suppresses inflammation and oxidative stress.	Zhu et al. (2017)
Rosmarinic acid	Polyphenols	C_18_H_16_O_8_	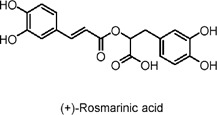	*Rosmarinus officinalis* L	High-fat diet (HFD)-fed C57BL/6 mice	50 mg/kg/day and 100 mg/kg/day (mice)	AMPK activation, enhancement of RCT (reverse cholesterol transport), upregulation of ABCG5/8 and CYP7A1	Reduces body weight, decreases plasma and hepatic cholesterol/triglycerides, promotes cholesterol excretion via the liver, increases fatty acid β-oxidation, and improves glucose homeostasis.	Nyandwi et al. (2021)
Ellagic acid	Polyphenols	C_14_H_6_O_8_	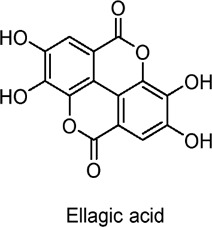	*Terminalia arjuna* (leaves)	3T3-L1 adipocytes, C2C12 myotubes	100 nM (*in vitro* for glucose transport assays)	AMPK activation, ERK, and atypical PKC (aPKC) activation	Increases glucose uptake, stimulates GLUT4 translocation, improves glucose homeostasis, does not affect Akt, enhances AMPK-ERK-aPKC pathway, potentially beneficial in managing insulin resistance.	Poulose et al. (2011)
Gallic acid	Polyphenols	C_7_H_6_O_5_	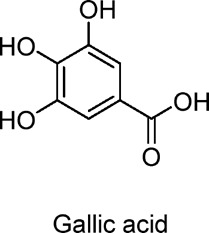	Found in tea, berries, fruits, and plants	Diet-induced obese mice, HepG2 cells	10 mg/kg (mice), 50 µM (HepG2 cells)	AMPK activation, Sirt1/PGC1α pathway, autophagy	Reduces body weight, improves glucose and insulin homeostasis, enhances mitochondrial function, reduces fat accumulation, increases thermogenesis (via UCP1), promotes fatty acid oxidation and autophagy.	Doan Khanh V et al. (2015)
Curcumin	Polyphenols	C_21_H_20_O_6_	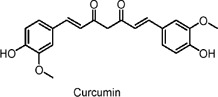	*Curcuma longa* (turmeric)	HepG2 cells, db/db diabetic mice, hepatoma cell lines (Hep3B, H4IIE)	50 µM (*in vitro*), 0.75% of diet (*in vivo*)	AMPK activation, NF-κB suppression, PPARγ activation, inhibition of gluconeogenic genes (PEPCK, G6Pase)	Improves glucose and lipid metabolism, reduces inflammation, enhances insulin sensitivity, decreases hepatic glucose production, reduces fatty acid synthesis, and promotes fatty acid oxidation.	Jiménez-Flores et al. (2014)
					Hep3B human hepatoma cells, H4IIE rat hepatoma cells	2–50 µM (*in vitro*)	AMPK activation, inhibition of gluconeogenic genes (PEPCK, G6Pase), ACC phosphorylation	Suppresses hepatic glucose production, reduces gluconeogenesis, enhances fatty acid oxidation, does not affect insulin signaling or glucose uptake, potential glucose-lowering and anti-diabetic effects.	Kim et al. (2009)
					Isolated rat hearts, neonatal rat cardiomyocytes, myocardial insulin resistance (IR) injury model	1 µM (*in vitro*), 200 mg/kg/day (oral, *in vivo* for 10 days)	SIRT1 activation, FOXO1 deacetylation, Bcl2 upregulation, Bax downregulation, increased mitochondrial antioxidant defenses	Attenuates oxidative mitochondrial damage, reduces myocardial infarction size, improves post-ischemic heart function, decreases apoptosis and oxidative stress, enhances SIRT1 signaling.	Yang et al. (2013)
Salidroside	Polyphenols	C_14_H_20_O_7_	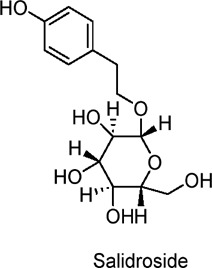	*Rhodiola rosea* L	db/db diabetic mice, primary mouse hepatocytes	25, 50, 100 mg/kg/day (*in vivo*); 0.1–10 µM (*in vitro*)	AMPK activation, PI3K/Akt/GSK3β pathway modulation, suppression of gluconeogenesis	Improves glucose uptake, reduces blood glucose and insulin levels, alleviates insulin resistance, enhances mitochondrial function, reduces lipid accumulation, ameliorates liver steatosis.	Zheng et al. (2015)
Resveratrol	Polyphenols	C_14_H_12_O_3_	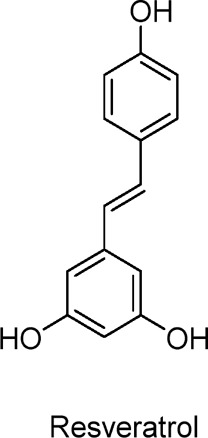	*Polygonum cuspidatum*, Grapes,Red wine	C2C12 myotubes (skeletal muscle cells)	25–100 µM (*in vitro*)	AMPK activation, ACC phosphorylation, PI-3 kinase/Akt pathway modulation	Increases glucose uptake in muscle cells, activates AMPK, promotes GLUT4 translocation, enhances insulin sensitivity, stimulates glucose metabolism, potential anti-diabetic effect.	Park et al. (2007)
					L6 myotubes (skeletal muscle cells)	25 µM (*in vitro*)	AMPK activation, mTOR and p70 S6K inhibition, IRS-1 dephosphorylation, Akt and GLUT4 translocation restoration	Restores insulin sensitivity, enhances glucose uptake, reverses palmitate-induced insulin resistance, reduces IRS-1 serine phosphorylation, activates AMPK, improves GLUT4 translocation.	Den Hartogh et al. (2020)
Dihydromyricetin	Flavonoids	C_15_H_12_O_8_	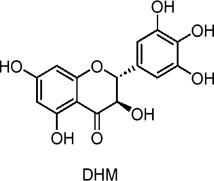	Ampelopsis grossedentata	High-fat diet (HFD)-fed C57BL/6J mice, SIRT3 knockout (KO) mice, MNK3 cells	300 mg/kg/day (*in vivo*), 10 μM (*in vitro*)	SIRT3 activation, IL-22 secretion from ILC3 cells, enhanced mitochondrial function and intestinal barrier integrity	Improves glucose tolerance, reduces hyperglycemia, enhances IL-22 production, restores intestinal barrier integrity, promotes mitochondrial function via SIRT3, and reduces insulin resistance in HFD mice.	Zhou Jie et al. (2022)
Baicalin	Flavonoids	C_21_H_18_O_11_	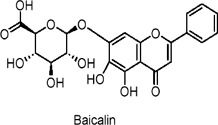	*Scutellaria baicalensis* (root)	High-fat diet (HFD)-fed C57BL/6J mice, C3H10T1/2 adipocytes	100 mg/kg/day (mice), 50 µM (*in vitro* for adipocytes)	AMPK activation, PGC1α activation, UCP1 expression	Promotes browning of white adipose tissue (WAT), activates brown adipose tissue (BAT), increases mitochondrial biogenesis, improves insulin sensitivity, enhances thermogenesis, and reduces obesity in mice.	Zhang et al. (2022)
				*Scutellaria baicalensis* (root)	High-fat diet (HFD)-induced obese C57BL/6J mice, 3T3-L1 adipocytes	50 mg/kg/day (mice), 100–400 µM (*in vitro* for adipocytes)	AKT/GLUT4 pathway activation, increased GLUT4 translocation, upregulation of PGC-1α, reduction of p38 MAPK and ERK phosphorylation	Increases insulin sensitivity, enhances glucose uptake, promotes GLUT4 translocation to plasma membranes, decreases body weight and adipose tissue mass, improves glucose tolerance, reduces HOMA-IR index.	Fang et al. (2018)
					High-fat diet (HFD)-fed Sprague-Dawley rats, HepG2 hepatoma cells	80 mg/kg/day (rats), 5–10 μmol/L (*in* *vitro*)	AMPK activation, ACC phosphorylation, inhibition of SREBP-1c, FAS	Reduces body weight, decreases visceral fat mass, improves serum lipid profile (reduces cholesterol, NEFA, TNF-α), decreases liver lipid accumulation, improves hepatic steatosis, and lowers insulin levels.	Guo et al. (2009)
				*Psoralea corylifolia* (seeds)	3T3-L1 adipocytes, C2C12 myoblasts	0.5–10 µM (*in vitro*)	Insulin signaling, AMPK activation, Akt phosphorylation, GLUT4 translocation	Improves glucose uptake, stimulates GLUT4 translocation, enhances insulin sensitivity, promotes adipocyte differentiation and lipid accumulation, increases adiponectin secretion, potentially beneficial for type 2 diabetes management.	Lee et al. (2016)
Quercetin, Quercetin-3-O-glycosides	Flavonoids	C_15_H_10_O_7_	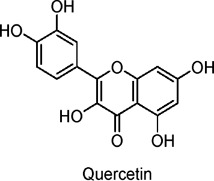	Plant-derived (natural)	Streptozotocin (STZ)-induced diabetic Wistar rats	100 mg/kg/day (oral administration for 15 days)	Reduces oxidative stress (MDA), increases GSH, affects cytokine levels (TNF-α, IL-6)	Decreases malondialdehyde (MDA) levels, increases glutathione (GSH), reduces triglycerides, LDL, total cholesterol, increases insulin levels, and positively affects cytokines (TNF-α, IL-6) in diabetic rats.	Do et al. (2018)
				*Vaccinium vitis-idaea* (lingonberry)	C2C12 myotubes (skeletal muscle cells), isolated mitochondria from Wistar rats	25–100 µM (*in vitro*)	AMPK activation, ACC phosphorylation, mild inhibition of mitochondrial ATP synthase	Increases glucose uptake, enhances AMPK activation, promotes energy metabolism, mildly inhibits mitochondrial respiration (reduces ATP synthesis), improves glucose transport independent of insulin.	Eid et al. (2010)
				Onion peel extract	High-fat diet/streptozotocin (HFD/STZ)-induced diabetic Sprague-Dawley rats	100 mg/kg/day (oral)	Upregulation of insulin receptor (INSR) and GLUT4, reduced oxidative stress, reduced inflammatory cytokines (TNF-α, IL-6)	Improves glucose tolerance, increases glycogen storage in liver and muscle, reduces blood glucose levels, decreases free fatty acids, reduces oxidative stress (MDA levels), and decreases inflammation (TNF-α, IL-6).	Kim et al. (2011)
Naringin	Flavonoids	C_27_H_32_O_14_	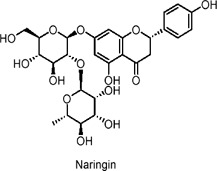	*Citrus changshanensis* (Huyou fruit)	HepG2 human hepatoma cells	0.2–5 μg/mL (*in vitro*)	AMPK activation, enhanced GLUT4 translocation	Increases glucose consumption, improves insulin sensitivity, reduces blood glucose, promotes glucose uptake in HepG2 cells via activation of the AMPK pathway, indicating hypoglycemic potential.	Zhang et al. (2012)
Genistein	Flavonoids	C_15_H_10_O_5_	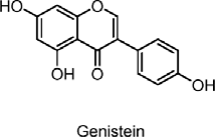	*Glycine* max (soybean)	INS1 cells, human pancreatic islets, streptozotocin (STZ)-induced diabetic mice	1–5 µM (*in* *vitro*), 0.25 g/kg diet (*in vivo*)	Activation of cAMP/PKA and ERK1/2 pathways, cyclin D1 upregulation, inhibition of β-cell apoptosis	Promotes pancreatic β-cell proliferation, improves glucose tolerance, increases insulin secretion, preserves β-cell mass, reduces β-cell apoptosis, prevents insulin-deficient diabetes.	Fu et al. (2010)

### Activation of AMPK by natural products

#### Natural products regulate AMPK at the molecular level

##### Direct action on AMPK molecules

Some natural products can activate AMPK at the molecular level through alterative regulation. For example, Berberine can specifically bind to the γ subunit of AMPK. Under normal physiological conditions, the intracellular ratio of AMP, ADP and ATP affects AMPK activity. When AMP levels are elevated, AMP binding to the γ subunit of AMPK causes a conformational change that activates AMPK. Berberine binds to and induces a conformational change in AMPK in a similar manner to AMP, making the active site of AMPK more susceptible to phosphorylation modification and thus activating AMPK (Hassanein et al., 2022). A number of natural products have important effects on the phosphorylation modification of AMPK. Natural polyphenols like resveratrol, which may affect AMPK phosphorylation by regulating upstream kinases. In cells, LKB1 is a common AMPK upstream kinase that phosphorylates key sites on the α-subunit of AMPK (e.g., Thr-172) to activate AMPK. Resveratrol can enhance the connection between LKB1 and AMPK, promote the phosphorylation process of AMPK by LKB1, and achieve the activation of AMPK at the molecular level (Tabrizi et al., 2022).

##### Regulation of related signaling molecules affects AMPK

Regulation of mTOR signaling molecules by natural products such as curcumin at the molecular level can affect AMPK. mTOR is a key serine/threonine kinase that has an important role in the regulation of cellular metabolism. There is a reciprocal regulatory relationship between mTOR and AMPK, and activation of mTOR inhibits AMPK. Curcumin inhibits mTOR activity and activates AMPK by inhibiting the inhibitory effect of mTOR on AMPK. This regulation is achieved by directly or indirectly affecting the active center of the mTOR molecule or its interaction with other signaling molecules (Salucci et al., 2023).

Ophiopogonin-B (OP-B) is an example of a natural product that regulates molecules associated with the PI3K - AKT signaling pathway and thus affects AMPK. In the PI3K-AKT signaling pathway, the activity of AKT has an inhibitory effect on AMPK. OP-B activates AMPK at the molecular level by inhibiting the activity of PI3K, thereby reducing the level of phosphorylation of AKT and decreasing the inhibitory effect of AKT on AMPK (Yuan et al., 2022). Similarly, malonyl-ginsenosides from *Panax ginseng* C.A.Mey (PG-MGR) treatment significantly reduced fasting blood glucose (FBG), triglyceride (TG), total cholesterol (TC), and low-density lipoprotein cholesterol (LDL-C) levels, and improved insulin resistance and glucose tolerance. In addition, protein expression levels of p-PI3K/PI3K, p-AKT/AKT, p-AMPK/AMPK, p-ACC/ACC, and GLUT4 were significantly upregulated in liver and skeletal muscle after PG-MGR treatment (Wang D.-S et al., 2022).

### Phenols

Phenolic compounds can positively influence diabetes-related metabolic processes by activating AMPK. One example is eugenol, a phenolic compound found in clove (Eugenia caryophyllata Thunb.), which has been investigated as a potential anti-diabetic agent. Jeong et al. (2014) reported that eugenol increases AMPK activity by enhancing the phosphorylation of its upstream kinase, CAMKK, in human hepatoma cell lines, primary rat hepatocytes, and in the livers of C57BL/6J mice fed a high-fat diet (HFD). This activation inhibited the HFD-induced changes in the nuclear CRTC2⋅CREB complex and reduced hepatic glucose production by suppressing the expression of gluconeogenic enzymes. Furthermore, another phenolic compound, [6]-gingerol, has been shown to enhance glucose uptake, activate AMPK phosphorylation, promote GLUT4 translocation, and improve insulin sensitivity (Li et al., 2024; Roufogalis, 2014; Song et al., 2017; De Lima et al., 2018; Momtaz et al., 2019).

### Polyphenols

Polyphenols are a type of phenols and belong to a specific type of phenolic compound found in many plants.Polyphenols are increasingly recognized for their significant role in regulating blood sugar levels, making them valuable in the prevention and management of T2DM. Generally, polyphenols activate AMPK by elevating AMP levels, often by interfering with mitochondrial ATP production. This interference occurs through various mechanisms, such as inhibiting Complex I, which blocks the respiratory chain (e.g., , berberine), inhibiting ATP synthase (Complex V) (e.g., , resveratrol), or acting as an uncoupling agent (e.g., , curcumin) (Momtaz et al., 2019). Notably, metabolites like galegine (derived from *Galega officinalis* L.) and octreotide activate AMPK by inhibiting Complex I in the mitochondrial respiratory chain, sharing a mechanism similar to that of resveratrol, EGCG (epigallocatechin-3-gallate epigallocatechin-3-gallate), and curcumin. These metabolites not only disrupt mitochondrial function and increase ADP/AMP levels but also selectively inhibit mitochondrial F1F0-ATPase/ATP synthase, further amplifying AMPK activation. EGCG in green tea lowered insulin resistance induced by dexamethasone, and improved the glucose uptake in rat L6 skeletal muscle cells. More than that, EGCG activates the PI3K/Akt pathway and enhances the phosphorylation of AMPK, and promotes GLUT4 translocation. Lui et al. (Golovinskaia and Wang, 2023) conducted a meta-analysis in seventeen trials comprising a total of 1,133 subjects and found that green tea intake significantly decreased the glycated hemoglobin and fasting glucose. This enhances their potential in diabetes treatment by offering new perspectives and strategies (Momtaz et al., 2019). Moreover, the versatility of polyphenols extends beyond mitochondrial regulation to potentially influencing various intracellular signaling pathways, broadening their therapeutic prospects in T2DM management.

Chlorgenic acid, a key Polyphenols found in coffee beans, positively regulates glucose metabolism. In HepG2 hepatocyte models, long-term exposure to chlorogenic acid has been shown to activate the AMPK signaling pathway (Ong et al., 2013). This activation promotes the translocation of the GLUT4 transporter, substantially increasing glucose uptake by the cells (Ong et al., 2013). The effectiveness of chlorogenic acid in these processes is directly linked to its ability to downregulate glucose-6-phosphatase (G6Pase). By inhibiting this enzyme, chlorogenic acid reduces glucose production and fatty acid synthesis in the liver, effectively managing blood glucose levels and fat metabolism (Ong et al., 2012; Ong et al., 2013). Several clinical trials have shown that green coffee extract and CGA improve hyperglycaemia and insulin resistance. One of these, a three-way randomised crossover study in healthy subjects, showed that chlorogenic acid in coffee reduced glucose absorption (Golovinskaia and Wang, 2023).

In addition, these natural products hold promise for more extensive research into their activation of AMPK and their effects on diabetes-related metabolic processes. For cichoric acid, its effects in different animal models warrant further exploration, as does its therapeutic potential when combined with other drugs for the treatment of diabetes and liver disease (Zhu et al., 2017). For instance, investigating the synergistic effects of chicoric acid in combination with insulin sensitizers or other hypoglycemic agents could offer additional options for clinical therapy (Sadeghabadi et al., 2019).

Rosmarinic acid originates from *Rosmarinus officinalis* L. Rosmarinic acid conspicuously reduced the body weight, blood glucose, plasma total cholesterol and triglyceride levels in C57BL/6J mice with high-fat diet (HFD)-induced hyperlipidemia (Nyandwi et al., 2021). Rosmarinic acid conspicuously reduced the body weight, blood glucose, plasma total cholesterol and triglyceride levels in C57BL/6J mice with HFD-induced hyperlipidemia (Nyandwi et al., 2021). Rosmarinic acid promotes fatty acid oxidation via AMPK-mediated induction of CPT1a (Nyandwi et al., 2021). This indicates the potential therapeutic implications of rosmarinic acid for dyslipidemia and derived metabolic disorders *in vivo* mouse models.

Ellagic acid, a naturally occurring polyphenol found primarily in berries and nuts in the form of ellagitannins, has been shown to enhance glucose uptake in 3T3-L1 adipocytes and C2C12 myotubes by activating AMPK-mediated GLUT4 translocation (Poulose et al., 2011).

The study of gallic acid can be extended to examine the variations in its effects on IR and lipid metabolism across different tissues and organs (Doan Khanh V et al., 2015). For example, its protective effects on the heart, kidneys, and other organs in diabetic conditions, as well as its regulatory influence on the relationship between gut microbiota and metabolic health, should be investigated. Additionally, the differences in the efficacy of gallic acid across various age groups and genders can also be explored.

Curcumin, a polyphenol, modulates the expression and signaling pathways of diabetes- and obesity-related genes. It improves muscle insulin resistance through the LKB1/AMPK pathway, downregulates liver gluconeogenesis genes, and stimulates SIRT1 (Sirtuin 1) (Picard and Auwerx, 2002; Kim et al., 2009; Na et al., 2011; Yang et al., 2013; Jiménez-Flores et al., 2014).

Similarly, Salidroside—another polyphenol derived from the roots of *Rhodiola rosea L*.—has shown dose-dependent efficacy in reducing hyperglycemia in db/db mice (Zheng et al., 2015). This compound lowers serum insulin levels, improves insulin sensitivity, and exerts an effect at 100 mg/kg/day comparable to 200 mg/kg/day of metformin *in vitro*. Mechanistically, Salidroside enhances the phosphorylation of AMPK, PI3K/Akt, and GSK3β in HepG2 cells (Zheng et al., 2015), thereby inhibiting hepatic gluconeogenesis through reduced AMPK-mediated expression of PEPCK and G6Pase. Salidroside-induced AMPK activation also leads to ACC phosphorylation, which helps diminish lipid accumulation in central nervous system tissues.

Further studies with isolated mitochondria reveal that Salidroside inhibits respiratory chain complex I, disrupts oxidative phosphorylation coupling, and causes moderate mitochondrial membrane depolarization, resulting in a transient rise in the AMP/ATP ratio. These mitochondrial effects likely underlie Salidroside’s anti-diabetic properties by activating the mitochondria-associated AMPK/PI3K/Akt/GSK3β pathway and enhancing metabolic flux (Zheng et al., 2015). In differentiated L6 rat muscle cells, Salidroside also promotes glucose uptake and increases AMPK and ACC phosphorylation in a dose-dependent manner (Li et al., 2008).

Hesperidin in combination with resveratrol as a Glo-1 inducer has been reported to improve metabolic and vascular health in overweight and obese human subjects, with a 25% reduction in fasting blood glucose and an increase in oral glucose sensitivity index of 42 mL/(min-m^2^) (Xiao et al., 2024). The polyphenol resveratrol is a non-flavonoid stilbene found in tiger orchid, red wine, and grapes. Research by Park et al. (2007) demonstrated that resveratrol inhibits cAMP-dependent phosphodiesterases (PDEs), leading to elevated levels of cAMP. Increased cAMP activates the cAMP-effector protein Epac1, which subsequently raises intracellular Ca^2+^ levels. This rise in Ca^2+^ stimulates the CaMKK-AMPK pathway through phospholipase C and ryanodine receptor-mediated Ca^2+^ release. Consequently, by increasing the phosphorylation of AMPK in insulin-treated C2C12 skeletal muscle cells, resveratrol enhances glucose uptake and improves insulin sensitivity. In another study, exposure of L6 skeletal muscle cells to palmitate, which models the elevated free fatty acid (FFA) levels associated with obesity and IR, resulted in increased serine phosphorylation of IRS-1 (insulin receptor substrate) and mTOR, while heightened phosphorylation and activation of p70 S6K (Den Hartogh et al., 2020). This condition also displaced insulin-stimulated GLUT4 membranes and reduced glucose uptake (Den Hartogh et al., 2020). However, resveratrol treatment reversed these effects, restoring insulin-stimulated glucose uptake, GLUT4 levels on the plasma membrane, and Akt phosphorylation/expression. Additionally, resveratrol significantly activated AMPK, suggesting its potential to mitigate fatty acid-induced IR in muscle cells. Resveratrol also promoted the phosphorylation of AMPK and its downstream target ACC in HepG2 hepatocytes (Zang et al., 2006). It has been suggested that liver AMPK inactivation is essential in diabetic hyperlipidemia, and AMPK activation could alleviate hyperlipidemia and atherosclerosis in diabetic mice. Um et al. found that after 13 weeks on a high-fat diet, resveratrol (400 mg/kg bw) greatly improved physical endurance, glucose tolerance, insulin sensitivity, mitochondrial biogenesis, and overall health in the experimental group (Um et al., 2010). Mice with AMPKα1 or AMPKα2 knocked out on the same diet did not exhibit these effects, indicating that AMPK mediates the beneficial effects of resveratrol (Um et al., 2010). Resveratrol’s effects on individuals with T2DM and obesity have been widely studied (Timmers et al., 2011). Research has shown that obese individuals who took 150 mg of resveratrol orally once a day for 30 days experienced improved glucose homeostasis and insulin sensitivity. This effect was achieved through the activation of AMPK, PGC-1α, and SIRT1 in muscle, increasing citric acid synthetase activity and improving mitochondrial efficiency. Resveratrol also enhances muscle mitochondrial respiration of substrates derived from fatty acids, mimicking the metabolic benefits of caloric restriction (Timmers et al., 2011). Moreover, the mechanism of resveratrol’s action is still being explored. Some studies suggest that resveratrol may improve metabolic health by modulating gut microbiota (Kasprzak-Drozd et al., 2024). The composition of gut flora is closely linked to metabolic diseases such as diabetes and obesity. Resveratrol may improve the intestinal microecological environment by promoting the growth of beneficial bacteria and inhibiting harmful bacteria, thereby influencing systemic metabolic processes.

In clinical settings, while some studies have found resveratrol to be beneficial for patients with type 2 diabetes and obesity, additional large-scale and long-term trials are necessary to verify both its safety and efficacy (Sun et al., 2022). For example, further studies could be conducted on the effects of different doses and dosing modalities on resveratrol’s efficacy, as well as its effects when combined with other drugs or treatments.

### Flavonoids

Many flavonoids exhibit potential anti-diabetic effects, particularly due to their antioxidant properties. Ninety-three postmenopausal women with T2DM intake 27 g flavonoid-enriched chocolate (850 mg flavan-3-ols and 100 mg isoflavones) per day showed a significant reduction in insulin resistance and improvement in lipid profiles compared to the placebo group, as demonstrated in a 1-year double-blind, randomized, controlled trial (Golovinskaia and Wang, 2023). Dihydromyricetin is a well-known flavonoids that has been shown to stimulate autophagy through the AMPK signaling pathway, thereby enhancing insulin sensitivity and correcting dysregulation of insulin secretion (Zhou Jie et al., 2022). Specifically, administration of dihydromyricetin (6 g/kg) alleviates high-fat diet-induced IR and abnormal insulin secretion, improving hyperglycemia by activating ILC3 cells. This effect is associated with the downregulation of SIRT3(Silent Mating-Type Information Regulation 2 Homolog-3), a key regulator of IR in T2DM, with PGC-1α (PPAR Coactivator-1α) levels significantly reduced in the muscle tissue of diabetic mice. Additionally, PGC-1α has been shown to enhance SIRT3 activity in hepatocytes and muscle cells, acting as an endogenous regulator of SIRT3. Thus, dihydromyricetin may regulate SIRT3 expression through the AMPK-PGC-1α pathway (N. Suganya et al., 2016).

Regarding baicalin’s role in different tissues, its effects on other metabolically active tissues, such as muscle and pancreas, warrant further investigation. In muscle tissue, studies could focus on whether baicalin enhances glucose uptake and fatty acid oxidation through the AMPK/PGC-1α pathway, thereby improving the energy metabolism efficiency of muscle (Zhang et al., 2022). In the pancreas, research could explore its regulatory effects on islet cell function and its influence on insulin secretion (Guo et al., 2009; Wang et al., 2017; Fang et al., 2018).

Flavonoids possess potential anti-diabetic effects, primarily through the activation of the AMPK signaling pathway and interactions with other pathways. Dihydromyricetin stimulates autophagy, potentially regulating SIRT3 expression via the AMPK-PGC-1α pathway to enhance insulin sensitivity (N. Suganya et al., 2016; Zhou Jie et al., 2022). Bavachin promotes the expression of lipogenic transcription factors and adiponectin secretion, improving insulin sensitivity through the Akt(Protein Kinase B) and AMPK pathways (Lee et al., 2016). Baicalin and its derivatives lower glucose levels, promote GLUT4 translocation, alleviate glucose intolerance, and reduce liver steatosis, warranting further investigation into their mechanisms of action, effects in various tissues, and potential clinical applications (Guo et al., 2009; Wang et al., 2017; Fang et al., 2018; Zhang et al., 2022; Eda et al., 2018). Quercetin enhances the phosphorylation of AMPK and its downstream effectors, inhibits glucose-6-phosphatase activity, promotes glucose uptake, and alleviates IR (Hardie, 2011; Kim et al., 2011; Dai et al., 2013; Gasparrini et al., 2016; Haddad and Eid, 2017; Do et al., 2018; Jayachandran et al., 2019; Zhang et al., 2021; Gnoni et al., 2022; Yan et al., 2023; Chen et al., 2024). Anthocyanin extracts activate AMPK, leading to the inhibition of fatty acid synthesis (Chang et al., 2013; Herrera-Balandrano et al., 2021; Liu et al., 2021; Feng et al., 2022; Jia et al., 2022).

The formation and promotion of fatty acid oxidation, along with the regulation of genes related to mitochondrial function, are crucial for metabolic control. Extracts from *Lonicera caerulea* L., *Plantago* L. 3-O-glucoside, *Oryza sativa L.*, and *Vaccinium uliginosum* L. anthocyanin have been shown to reduce lipid accumulation in adipocytes, inhibit the expression of liver gluconeogenic genes, and promote glucose uptake, thereby alleviating hyperglycemia and hyperlipidemia (Herrera-Balandrano et al., 2021; Liu et al., 2021; Feng et al., 2022; Jia et al., 2022). In human clinical trials, *V. uliginosum* L. decrease cardiovascular risk factors in obese men and women with metabolic syndrome, and improved insulin sensitivity was observed (Hameed et al., 2020). Naringin enhances insulin sensitivity and reduces blood glucose levels via the AMPK pathway, while genistein promotes insulin secretion, stimulates glucose uptake, and lowers non-fasting glucose levels, partly through the AMPK pathway (Fu and Liu, 2009; Lee et al., 2009; Fu et al., 2010; Ha et al., 2012; Zhang et al., 2012). A randomized, double-blind, placebo-controlled clinical trial included 54 postmenopausal women with T2DM, who intake two genistein capsules (108 mg genistein) daily. After 12 weeks of intervention, fasting blood glucose is lowered when compared with the placebo group (Braxas et al., 2019). Future studies should further investigate the relationship between flavonoids and AMPK to better understand their anti-diabetic effects.

### Terpenoids

Terpenes and steroids represent a broad class of plant metabolites synthesized via the mevalonate pathway. These metabolites undergo conversion into isoprene units, which are subsequently utilized in different biosynthetic pathways to form monoterpenes, sesquiterpenes, diterpenes, triterpenes, or steroids. Several of these metabolites have been studied for their potential therapeutic applications. Table 2 provides an overview of recent research on terpenoids and alkaloids derived from natural products, demonstrating their potential as anti-diabetic agents, primarily through the activation of AMPK.

**TABLE 2:** Other natural products with AMPK activsting propertis

Natural components	Classification	Molecular formula	Chemical structure	Source	Test models	Dose	Mechanisms	Specific effects	Refs
Atractylodes macrocephala I (AT-I), Atractylodes macrocephala II (AT-II)	Terpenoids	C_15_H_18_O_3_, C_15_H_18_O_4_	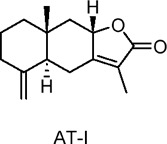 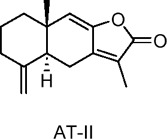	*Atractylodes macrocephala* Koidz	C2C12 myotubes (skeletal muscle cells)	50 μg/mL (*in vitro*)	AMPK and PI3K/Akt activation, GLUT4 translocation	Increases glucose uptake, enhances GLUT4 translocation to the plasma membrane, improves insulin sensitivity, ameliorates TNF-α-induced insulin resistance, stimulates glucose metabolism​	Chao et al. (2016)
Atractylenolide III (AIII)	Terpenoids	C_15_H_20_O_3_	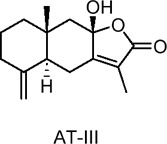	*Atractylodes macrocephala* Koidz	C2C12 myotubes (mouse skeletal muscle cells)	10, 20, 50 µM (*in* *vitro*)	AMPK activation, SIRT1 and PGC1α upregulation, mitochondrial biogenesis, glucose uptake enhancement	Increases glucose uptake, enhances mitochondrial biogenesis, boosts ATP production, improves energy metabolism by activating AMPK, SIRT1, and PGC1α, promotes insulin sensitivity​	Song et al. (2017)
Carnosic acid	Terpenoids	C_20_H_28_O_4_	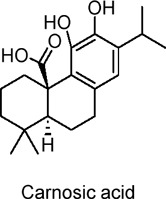	*Rosmarinus officinalis* (rosemary)	HepG2 hepatocytes, C2C12 myotubes, HEK293T cells	10 µM (HepG2 cells, 24 h)	AMPK activation, suppression of G6PC and PCK1 gene expressions	Suppresses gluconeogenesis, inhibits lipogenic genes (FAS, ACC1, SREBP-1c), enhances fatty acid oxidation (via PGC-1α and CPT1A), reduces cell proliferation, promotes apoptosis (via p53 and caspase-3), potentially protects against diabetes, fatty liver, and hepatoma	Hasei et al. (2021)
Platycodin D	Terpenoids	C_58_H_94_O_27_	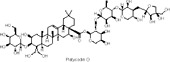	*Platycodon grandiflorum* (root)	High-fat diet (HFD)/streptozotocin (STZ)-induced type 2 diabetic mice	2.5 mg/kg and 5 mg/kg (*in vivo*)	AMPK activation, downregulation of PCK1, G6Pase, ACC phosphorylation, CPT-1 upregulation	Reduces hyperglycemia, improves glucose tolerance and insulin sensitivity, lowers serum cholesterol and triglycerides, reduces hepatic fat accumulation, and improves liver function and injury​	Shen et al. (2023)
Saikosaponin A	Terpenoids	C_42_H_68_O_13_	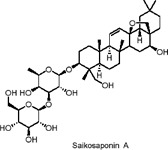	*Bupleurum chinense* DC. (root)	3T3-L1 adipocytes (*in vitro*)	0.938–15 µM (*in vitro*)	AMPK activation, MAPK pathway inhibition (ERK, p38), downregulation of PPARγ and C/EBPα	Inhibits lipid accumulation, suppresses adipogenesis by reducing transcription factors (PPARγ, C/EBPα), and decreases expression of lipogenic genes (FAS, LPL, adiponectin)​	Lim et al. (2021)
Saikosaponin D	Terpenoids	C_42_H_68_O_13_	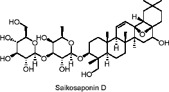	*Bupleurum chinense* DC. (root)	3T3-L1 adipocytes (*in vitro*)	0.938–7.5 µM (*in vitro*)	AMPK activation, MAPK pathway inhibition (ERK, JNK), downregulation of PPARγ and C/EBPα	Inhibits lipid accumulation, suppresses adipogenesis by reducing transcription factors (PPARγ, C/EBPα), and decreases expression of lipogenic genes (FAS, LPL, adiponectin)​	Lim et al. (2021)
Isoliensinine	Alkaloids	C_37_H_42_N_2_O_6_	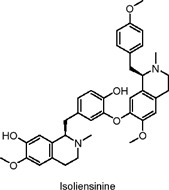	*Nelumbo nucifera* (lotus seed embryo)	KK-Ay diabetic mice, L6 myotubes (rat skeletal muscle cells)	30 mg/kg and 60 mg/kg (*in vivo*); 5–15 μg/mL (*in vitro*)	AMPK activation, increased GLUT4 translocation, downregulation of PPARγ, SREBP-1c, and ACC phosphorylation	Improves glucose uptake, reduces fasting blood glucose, decreases body weight, enhances insulin sensitivity, lowers triglycerides, cholesterol, and free fatty acids, reduces adipocyte size​	Yang et al. (2017)
Berberine	Alkaloids	C_20_H_18_NO_4_	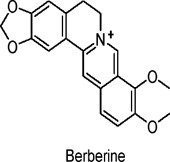	*Coptis chinensis* (Huanglian)	Human retinal Müller cells, *in vitro* study on oxidized LDL-induced damage in diabetic retinopathy (DR)	5 µM (*in vitro*)	AMPK activation, reduction of oxidative stress, inhibition of autophagy, apoptosis, angiogenesis, and inflammation	Protects Müller cells from oxidized LDL-induced damage, reduces cell death, oxidative stress, and inflammation, inhibits VEGF upregulation, mitigates diabetic retinopathy	Fu et al. (2016)
					db/db mice (obesity and type 2 diabetes model), 3T3-L1 adipocytes (*in vitro*)	75 mg/kg, 150 mg/kg (*in vivo*); 3 μM, 10 µM (*in vitro*)	AMPK activation, inhibition of TBK1/IKKε pathways, increased UCP1 expression, enhanced mitochondrial biogenesis	Reduces body weight, improves glucose tolerance, enhances lipid metabolism, decreases inflammation, improves insulin sensitivity, reduces fat accumulation, enhances energy expenditure​	Wang M et al. (2022)
Hirsutine	Alkaloids	C_22_H_28_N_2_O_3_	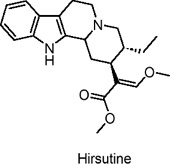	Uncaria rhynchophylla	High-fat diet (HFD)-induced diabetic mice, HepG2 and H9c2 cell models	5, 10, 20 mg/kg (*in vivo*); 0.1–10 µM (*in vitro*)	PI3K/Akt activation, AMPK/ACC pathway activation, GLUT4 upregulation, inhibition of gluconeogenesis	Improves insulin sensitivity, reduces hepatic steatosis and cardiac hypertrophy, increases glucose uptake, enhances glycogen synthesis, reduces lipid accumulation, and promotes glycolysis​	Hu et al. (2022)
Laurolitsine (LL)	Alkaloids	C_18_H_19_NO_4_	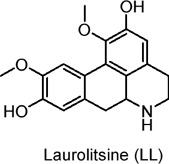	*Cynodon grandis* (bark extract)	db/db mice (type 2 diabetes model), HL-7702 hepatocytes	50, 100, 200 mg/kg (*in vivo*), 1.25–5 µM (*in vitro*)	LKB1/AMPK pathway activation, ADP/ATP ratio modulation, gut microbiota regulation	Reduces fasting blood glucose, improves insulin sensitivity and glucose tolerance, decreases lipid accumulation, protects liver, kidney, and pancreas, reduces inflammatory markers, modulates gut microbiota​	Yong et al. (2022)
Tanshinone IIA	Quinones	C_19_H_18_O_3_	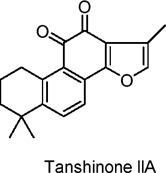	*Salvia miltiorrhiza* Bunge	C2C12 myotubes (mouse skeletal muscle cells)	10, 20, 50 µM (*in vitro*)	AMPK activation, SIRT1 and PGC1α upregulation, mitochondrial biogenesis, glucose uptake enhancement	Increases glucose uptake, promotes mitochondrial biogenesis, boosts ATP production, activates AMPK, enhances insulin sensitivity, improves energy metabolism in skeletal muscle​	Gong et al. (2009)
total lignans from burdock (TLFA)	Phenylpropanoids	C_22_H_22_O_8_	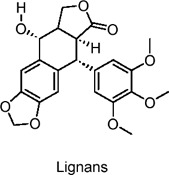	*Arctium lappa L.* (Great burdock fruit)	ob/ob mice (type 2 diabetes model), C57BL/6J mice, L6 myotubes, rat primary hepatocytes	25–200 mg/kg (*in vivo*), 1–10 µM (*in vitro*)	AMPK activation (via complex I inhibition), reduced mitochondrial respiration, GLUT4 translocation	Reduces fasting and postprandial blood glucose, improves insulin sensitivity, enhances glucose uptake, decreases gluconeogenesis and lipid synthesis, improves glucose tolerance, reduces lipid accumulation​	Huang et al. (2012)
					Goto-Kakizaki (GK) rats (spontaneous type 2 diabetes model), Wistar rats	300 mg/kg (*in vivo*), 50 mg/kg nateglinide as control	Insulin secretion stimulation, GLP-1 release promotion, α-glucosidase inhibition	Lowers fasting and postprandial blood glucose, improves glucose tolerance, reduces glycated hemoglobin, enhances insulin secretion, promotes GLP-1 release, inhibits glucose absorption​	Xu et al. (2014)
					ob/ob mice (type 2 diabetes model), L6 muscle cells, primary hepatocytes	200 mg/kg (*in vivo*), 1–10 µM (*in vitro*)	AMPK activation (indirect), inhibition of mitochondrial complex I, enhanced glucose uptake, reduced gluconeogenesis	Improves glucose tolerance, lowers fasting and postprandial glucose, enhances insulin sensitivity, decreases lipid synthesis, reduces subcutaneous fat without affecting visceral fat​	Miele and Beguinot (2012)
					Sedentary mice (endurance model), H9C2 (cardiac muscle cells), C2C12 (skeletal muscle cells)	8 mg/kg/day (*in vivo*), 1–40 µM (*in vitro*)	AMPK activation (via CaMKK and LKB1), PGC-1α upregulation, mitochondrial biogenesis, fatty acid oxidation	Enhances endurance capacity, increases mitochondrial function, upregulates fatty acid oxidation genes, improves aerobic capacity, increases treadmill endurance without exercise training​	Tang et al. (2011)
					KKAy mice (obese, type 2 diabetes model), C57BL/6J mice (normal control)	125 and 250 mg/kg (*in vivo*)	PI3K/Akt activation, AMPK signaling, insulin secretion, PTP1B inhibition, leptin reduction, adiponectin increase	Lowers fasting blood glucose, HbA1c, and body weight; improves glucose tolerance; reduces triglycerides, FFA, LDL-C; increases HDL-C; reduces adipocyte size and lipid accumulation​	Gao et al. (2018)
Spatholobus suberectus (formononetin-rich extract)				*Spatholobus suberectus* (stem)	db/db mice (type 2 diabetes, nephropathy model), BSA-AGEs *in vitro*	50 mg/kg/day (*in vivo*); 10–100 μg/mL (*in vitro*)	Inhibits AGEs formation and cross-linking, upregulates Nrf2 and Glo1, reduces RAGE expression	Improves lipid profile (lowers LDL, triglycerides, FFAs), reduces albumin-to-creatinine ratio, prevents AGEs accumulation, decreases oxidative stress, mitigates diabetic nephropathy	Do et al. (2018)

Sesquiterpene lactones extracted from the dried rhizome of *Atractylodes macrocephala* Koidz. Have been used for centuries in several Asian countries to treat digestive diseases and diabetes. Chao et al. investigated the effects of two sesquiterpene lactones, Atractylodes macrocephala I (AT-I) and Atractylodes macrocephala II (AT-II), on glucose uptake in C2C12 skeletal muscle cells of mice (Chao et al., 2016). The results showed that, without affecting GLUT1 levels, both metabolites significantly increased GLUT4 protein levels and facilitated its translocation to the plasma membrane. The activation of AMPK and PI3K(phosphatidylinositol 3-kinase)/Akt signaling pathways was linked to the observed enhancement in glucose uptake. Furthermore, Song et al. examined the effects of Atractylenolide III (AIII), a specific metabolite from the same species, on energy metabolism in C2C12 cells (Song et al., 2017). Their findings revealed that AIII significantly increased glucose uptake and upregulated the expression of mitochondrial biogenesis markers, including mitochondrial transcription factor A (TFAM) and nuclear respiratory factor-1 (NRF-1), as well as PGC1α. Additionally, AIII increased the expression of SIRT1, total ATP levels, AMPK phosphorylation, and mitochondrial mass. These results suggest that AIII may hold promise for treating T2DM by improving energy metabolism in skeletal muscle. In studies on diterpenoids, including coniferolactone, tanshinone IIA, and dehydroconiferolactone, the effects on glucose metabolism were examined. Pinusolide, extracted from *Biota orientalis L.* (*Cupressaceae*) in East Asia, was found to induce AMPK phosphorylation and stimulate glucose uptake via the LKB1-mediated AMPK activation pathway (Hwang et al., 2013). Research on carnosic acid, a terpenoid, can be extended to investigate its specific protective mechanisms against various types of hepatocellular carcinoma, as well as its long-term effects on the prevention and progression of liver cancer (Hasei et al., 2021). In addition, evaluating carnosic acid in combination with other antioxidants or anti-tumor drugs will help clarify its therapeutic potential and establish its role in comprehensive treatment regimens.

Meanwhile, in the context of blood glucose regulation, traditional medicine has utilized the pine plant *Abies balsamea* (L.) Mill, from which Nachar et al. isolated three active diterpenes—squalene, fire acid, and dehydrofire acid (Wen et al., 2005; Nachar et al., 2015). These metabolites reduce glucose-6-phosphatase activity and enhance glycogen synthetase via Akt and AMPK activation. Beyond Abies balsamea, other terpenoids and steroids have also been investigated for their anti-diabetic effects, often through the activation of AMPK. For instance, rosiglitazone has demonstrated significant anti-diabetic properties in multiple studies.A recent work examined tormentic acid’s influence on diabetes and dyslipidemia in high-fat-fed C57BL/6J mice, using rosiglitazone as a reference treatment (Wu et al., 2014). The work found that tormentic acid suppressed phosphoenolpyruvate carboxykinase (PEPCK), induced AMPK phosphorylation, and elevated both Akt phosphorylation and GLUT4 expression in skeletal muscle, thereby improving insulin sensitivity. It also increased phosphorylated AMPK in the liver, potentially reducing hepatic glucose production while boosting GLUT4 in skeletal muscle. Additionally, tormentic acid exhibited lipid-lowering activity by decreasing SREBP-1c and Apo C-III (Apolipoprotein C-III) levels in the liver while increasing PPAR-α expression, which in turn lowered blood triglyceride concentrations. Taken together, these findings indicate that tormentic acid holds promise for managing diabetes and its associated dyslipidemia (Wu et al., 2014).

Platycodin D, a triterpenoid saponin extracted from platycodon root, exhibits significant pharmacological activity. Shen et al. investigated the effects of Platycodin D in a mouse model of T2DM induced by a HFD and streptozotocin (STZ) (Shen et al., 2023). The study evaluated doses of 2.5 and 5.0 mg/kg of Platycodin D as dietary supplements for the treatment of T2DM. Both doses significantly reduced weight gain and fasting blood glucose (FBG) levels induced by the high-fat diet, improved glucose and insulin tolerance, and helped maintain glucose homeostasis. Regarding lipid metabolism, Platycodin D lowered systemic levels of total cholesterol (TC), triglycerides (TG), and high-density lipoprotein (HDL), and reduced hepatic lipid accumulation via the AMPK/ACC/CPT-1 fatty acid assimilation pathway. Molecular docking studies revealed that Platycodin D directly interacts with key glycolipid-metabolizing proteins, such as AMPK. Furthermore, Platycodin D enhanced energy metabolism in a mouse model of diabetic cardiomyopathy (DCM) by activating AMPK, increasing GLUT4 transporter expression, and stimulating autophagy-associated proteins. *In vitro* experiments demonstrated that Platycodin D improved cell viability, reduced the cytotoxic effects of palmitate and glucose on H9c2 cells, and activated AMPK protein expression. Additionally, the AMPK activator AICAR (5-aminoimidazole-4-carboxamide ribonucleoside) (1 mM) was shown to upregulate AMPK expression in H9c2 cells exposed to high glucose and fat conditions (Wang et al., 2024).

Saikosaponin A (SSA) and Saikosaponin D (SSD) are triterpenoid saponins extracted from *Bupleurum chinense* DC., known for their anti-inflammatory, antipyretic, and hepatoprotective effects. Studies have demonstrated that both SSA and SSD reduce the expression of several key transcription factors in 3T3-L1 preadipocytes, including PPARγ, C/EBP-α, SREBP-1c, and lipocalin (Lim et al., 2021). This downregulation led to the decreased expression of key adipogenesis-related genes such as fatty acid binding protein (FABP4), FAS, and lipoprotein lipase (LPL) Additionally, SSA and SSD inhibited the phosphorylation of p38 and ERK1/2(extracellular-regulated kinase 1/2) while enhancing the phosphorylation of AMPK and its downstream target, ACC (Lim et al., 2021). The activation of AMPK and MAPK (mitogen-activated protein kinase) pathways mediates the observed anti-lipogenesis effects during the early stages of adipocyte differentiation. Since this study is the first to confirm the anti-adipogenic effects of SSA and SSD, further research, including animal models and clinical trials, is necessary to assess their anti-obesity potential.

Malonyl-ginsenosides, predominantly found in fresh and air-dried *P. ginseng* C. A. Mey. roots, represent an isotype of ginsenosides characterized by the attachment of propanediol fragments to glucose molecules of neutral ginsenosides. Treatment with these ginsenosides resulted in significant reductions in FBG, TG, TC, and low-density lipoprotein cholesterol (LDL-C) levels, alongside improvements in insulin sensitivity and glucose tolerance (Wang D.-S et al., 2022). Additionally, malonyl-ginsenosides mitigated liver damage by reducing aspartate aminotransferase (AST) and alanine aminotransferase (ALT) levels. The treatment also significantly increased the expression of p-PI3K/PI3K, p-AKT/AKT, p-AMPK/AMPK, p-ACC/ACC, and GLUT4 proteins in liver and muscle tissues, while decreasing the levels of p-IRS-1/IRS-1, Fas, and SREBP-1c (Wang D.-S et al., 2022). These findings suggest that malonyl-ginsenosides promote glycolipid metabolism and alleviate IR by modulating the IRS-1/PI3K/Akt and AMPK signaling pathways (Wang M et al., 2022).

The role of terpenoids in bioactive triterpenes is particularly notable. Hu et al. investigated the effects of six terpenoids extracted from Salicylus DC. (Lardizabalaceae) on glucose uptake, using insulin-resistant HepG2 cells as a model. One of these metabolites, 3-O-α-L-arabinopyranosyl-(1→3)-α-L-rhamnopyranosyl-(1→2)-α-L-arabinopyranosyl-30-norhederagenin-28-oic acid, significantly improved glucose uptake and metabolism in liver cells (Hu et al., 2014). Furthermore, this saponin activated the IR/IRS-1/PI3K/Akt signaling pathway, enhancing AMPK phosphorylation (Hu et al., 2014).

### Alkaloids

The fruits of black mulberry (*Morus nigra* L.) and other mulberry species are rich in alkaloid metabolites with potential health benefits that have been widely studied. Eruygur et al. investigated 1-Deoxynojirimycin, a piperidine alkaloid isolated from black mulberry leaves, which acts as a potent alpha-glucosidase inhibitor (Eruygur and Dural, 2019). Due to its structural similarity to sugar, this alkaloid inhibits glucosidase activity. It has been shown to activate AMPK and Akt, while inhibiting NF-κB (nuclear factor kappa B) and MMP-2 activity, mechanisms that contribute to its potential anti-diabetic effects (Chan et al., 2013). Another plant with potential anti-diabetic properties is the lotus flower (*Nelumbo nucifera* Gaertn) (Kato et al., 2015). A 50% methanol-water extract from dried embryonic seeds of this plant demonstrated enhanced glucose uptake activity in L6 muscle cells, which is linked to its hypoglycemic effects. The active metabolite identified in the extract was phenylisoquinoline protoberberine 4′-O-β-D glycoside. Interestingly, this metabolite does not exert its hypoglycemic effects via AMPK or PI3K pathways, as many other agents do. Instead, it works by activating β2-adrenergic receptors, a mechanism involved in blood glucose regulation in insulin-resistant patients (Kato et al., 2015). Yang et al. isolated the alkaloid isoliensinine protoberberine from the same species and evaluated its effects using both *in vitro* (L6 cells) and *in vivo* (KK-Ay mice) models (Yang et al., 2017). After 4 weeks of *in vitro* treatment, the alkaloid increased GLUT4 translocation by 2.5-fold and elevated serum insulin levels *in vivo* by 24%. Moreover, it significantly reduced fasting blood glucose levels by approximately 50% and body weight by around 19% in the treated mice. The authors concluded that isoliensinine protoberberine alleviates T2DM-associated hyperlipidemia by regulating AMPK phosphorylation and promoting GLUT4 expression (Yang et al., 2017). Table 2 summarizes recent research on alkaloids in natural products as potential anti-diabetic agents through the activation of AMPK.

Berberine, an isoquinolone alkaloid, has shown favorable effects on glucose and lipid metabolism in animal and human studies. In a single-centre, randomised controlled study, administration of berberine (0.4 g 3 times daily) to patients with T2DM for 6 months improved glycated haemoglobin (HbA1c), blood urea nitrogen (BUN), systolic blood pressure (SP), and high-sensitivity C-reactive protein (hs-CRP) indices (Shrivastava et al., 2023). Berberine is a significant phytochemical known for its ability to activate AMPK (Zhang et al., 2020). This metabolite has substantial potential for regulating glycolipid metabolism, making it a promising candidate for the treatment of diabetes and obesity (Ren et al., 2023). In various diabetic rodent models, berberine has demonstrated numerous pharmacological effects, including reducing total cholesterol, LDL cholesterol, triglyceride levels, and body weight, as well as improving glucose tolerance and enhancing the expression of LDL and insulin receptor (Insr) genes (Day et al., 2017). These effects are largely attributed to the activation of AMPK, which regulates mitochondrial function and improves insulin sensitivity. Berberine induces phosphorylation at AMPK Thr172 (threonine172), and studies have shown that it strongly stimulates the α1 and α2 subunits of AMPK, resulting in a reduced intracellular energy state (Ren et al., 2023). While the involvement of CaMKKβ and LKB1 in AMPK activation has been discussed, it has been suggested that berberine’s effect on AMPK may not be entirely dependent on these pathways. Mitochondria, which are central to ATP production via oxidative phosphorylation and support numerous ATP-dependent metabolic processes (such as cholesterol synthesis, lipogenesis, gluconeogenesis, and extracellular matrix synthesis), are significantly affected by berberine. Research has shown that berberine inhibits mitochondrial respiratory complex I in the gut and liver, leading to decreased mitochondrial activity, which subsequently activates AMPK (Yu et al., 2021). This inhibition enhances glucose metabolism and suppresses lipid metabolism, including the uptake and synthesis of fatty acids in the gut and liver.

These conclusions are supported by a mouse model study that demonstrated berberine’s protective effects against obesity, diet-induced IR, and T2DM by inhibiting oxidative phosphorylation (Jin et al., 2017). Additionally, berberine prevented high-glucose-induced podocyte apoptosis in mice and restored autophagy defects caused by hyperglycemia (Jin et al., 2017). In human retinal cells, berberine was shown to inhibit LDL-induced cell death and dysfunction by activating AMPK (Fu et al., 2016). The use of AMPK inhibitor metabolite C partially but significantly reversed berberine’s effects, further confirming the critical role of AMPK in mediating its beneficial actions (Fu et al., 2016). However, berberine’s poor solubility and limited membrane permeability have resulted in an oral bioavailability of less than 1%, posing challenges to its clinical application (Wang D.-S et al., 2022). To overcome this issue, researchers developed a 1:1 eutectic mixture of berberine-ibuprofen (BJ) using pharmaceutical salt reduction and co-crystallization techniques. Pharmacokinetic studies revealed that the bioavailability of BJ is three times higher than that of berberine alone. Moreover, BJ inhibited TBK1 and activated AMPK, suggesting enhanced mitochondrial biogenesis. In addition to improving energy metabolism by blocking the IKKε/adrenergic/cAMP pathway, BJ also reduced fat accumulation, improved IR, and alleviated inflammatory responses in adipose tissue in db/db mice (Wang M et al., 2022). Furthermore, BJ improved glucose and lipid homeostasis (Wang D.-S et al., 2022). These findings indicate that BJ has the potential to significantly enhance the clinical application of berberine.

Hirsutine, an indole alkaloid extracted from broom, has demonstrated beneficial effects on impaired glucose tolerance, maintenance of liver glycogen content, and reduction of liver lipid accumulation in diabetic mice induced by a HFD (Hu et al., 2022). In HepG2 cells, Hirsutine downregulates the mRNA expression levels of key liver glucose isoenzymes, such as PEPCK and G6Pase, as well as transcription factors PGC-1α and FOXO1 (forkhead box protein O 1), leading to reduced glucose production (Hu et al., 2022). Additionally, Hirsutine-induced phosphorylation of ACC inhibits its activity, increasing mitochondrial fatty acid oxidation and, thereby, enhancing insulin sensitivity in H9c2 cells. Chronic activation of AMPK by Hirsutine is also associated with GLUT4 translocation in muscle tissue, which contributes to improved glucose regulation (Hu et al., 2022). Since GLUT4 expression is impaired in hyperglycemic and hyperinsulinemic conditions, the significant increase in GLUT4 expression induced by Hirsutine suggests that it improves insulin sensitivity in H9c2 cells via the AMPK pathway (Hu et al., 2022).

Laurolitsin (LL), the primary alkaloid in the bark of *Cynodon grandis* (CG), exhibits strong anti-diabetic effects. LL increases glucose utilization and activates AMPK without causing lactic acid accumulation or cytotoxicity. In db/db mice, LL has been shown to lower blood glucose levels, reduce body weight, improve insulin sensitivity, optimize lipid metabolism, and protect liver, kidney, and pancreatic function (Yong et al., 2022). Moreover, LL contributed to weight loss in these diabetic mice. Transcriptome analysis revealed that the hypoglycemic effect of LL is linked to the regulation of mitochondrial oxidative phosphorylation. Additionally, LL significantly activates the LKB1/AMPK pathway by inhibiting ATP production and altering the ADP/ATP ratio in hepatocytes and other tissues (Yong et al., 2022). These findings suggest that laurolitsin holds great promise for the treatment of diabetes, primarily through the activation of the AMPK pathway.

### Quinones

In studies on diterpenoids, including coniferolactone, tanshinone IIA, and dehydroconiferolactone, the effects on glucose metabolism were examined. Pinusolide, extracted from *Biota orientalis L.* (Cupressaceae) in East Asia, was found to induce AMPK phosphorylation and stimulate glucose uptake via the LKB1-mediated AMPK activation pathway (Hwang et al., 2013). Tanshinone IIA, extracted from *Salvia miltiorrhiza* Bunge, improved glucose tolerance, insulin sensitivity, and glucose metabolism by inhibiting the differentiation of 3T3-L1 preadipocytes and the transcriptional activity of PPARγ(peroxisome proliferator-activated receptor γ) (Gong et al., 2009). It also significantly reduced PERK (RNA-activated protein kinase-like ER resident kinase) and JNK (c-Jun-N-terminal kinase) phosphorylation while increasing AMPK activation, thereby enhancing insulin-mediated Akt phosphorylation. This suggests that tanshinone IIA may promote glucose uptake through AMPK, particularly in response to ER stress. Inhibition of AMPK or LKB1 via siRNA was shown to counteract the effects of tanshinone IIA. Additionally, tanshinone IIA reduced body weight and blood glucose levels in db/db mice without affecting food intake. These findings indicate that tanshinone IIA enhances AMPK activity, mitigating IR caused by ER(endoplasmic reticulum) stress and improving insulin sensitivity and glucose metabolism (Hwang et al., 2013).

### Other plant species

*Arctium lappa* L., a member of the Asteraceae family, possesses significant anti-diabetic and antioxidant properties. It has been shown to aid in the treatment of T2DM and its complications by improving blood glucose regulation and reducing IR. Huang and his colleagues discovered that burdock promotes the phosphorylation of AMPK (Huang et al., 2012). In a study using Goto-Kakizaki rats, administering 300 mg/kg of total lignans from burdock (TLFA) twice daily before meals for 12 weeks reduced fasting and postprandial blood glucose levels, improved HbA1c regulation, and enhanced glucose tolerance (Xu et al., 2014). The inhibition of alpha-glucosidase activity, thereby reducing postprandial hyperglycemia, was identified as an effective strategy for treating T2DM (Zhu et al., 2020; Proença et al., 2022). In an obese mouse model, arctigenin was found to improve glucose and lipid metabolism by reducing mitochondrial respiration, inducing AMPK activation, stimulating muscle glucose uptake, and inhibiting hepatocyte regeneration and adipogenesis (Huang et al., 2012; Miele and Beguinot, 2012). Additionally, arctigenin, a metabolite derived from celery, was shown to phosphorylate AMPK through CaMKK- and LKB1-dependent pathways in C2C12 and H9C2 cells, promoting glucose uptake, enhancing mitochondrial biogenesis, and increasing glycolysis (Tang et al., 2011). Gao and his team recently conducted a study using diabetic and obese KKAy rodent models (Gao et al., 2018). Over an 11-week period, the mice were administered TLFA at doses of 125 mg/kg and 250 mg/kg. The results demonstrated that TLFA reduced fasting blood glucose levels, glycated hemoglobin, body weight, serum triglycerides, and free fatty acids, while improving oral glucose tolerance and increasing HDL cholesterol. The study identified the stimulation of insulin secretion, activation of the PI3K/Akt pathway, and activation of the AMPK signaling pathway as key mechanisms (Gao et al., 2018). These findings underscore the potential of burdock and its bioactive metabolites as therapeutic agents for T2DM, with their effects being primarily mediated through the activation of AMPK and related metabolic pathways.

DoMH et al. report another related study on the antidiabetic activity of *Spatholobus suberectus* Dunn, focusing on the activation of the Akt-AMPK pathway (Do et al., 2018). Phosphorylated (p)-ERK, p-p38 MAPK and nuclear transcription factor-κB (NF-κB) p65 have been found to be at high expression in human diabetic nephropathy patients. Saphenous suberectus extract alleviated diabetes-induced kidney damage in db/db mouse models through activation of the Akt-AMPK pathway. This suggests that Saphenous suberectus may have a renal protective effect by modulating the AMPK signaling pathway, further supporting its potential as a therapeutic agent for diabetes. Besides, in preclinical studies, many natural products have recently been reported to alleviate kidney disease (Hu et al., 2023). For example, a prospective, single-centre randomised controlled trial was designed to study the efficacy and safety of the combination of tretinoin and angiotensin receptor blockers (ARBs) in the treatment of diabetic kidney disease (DKD). The primary endpoint was a reduction in 24-h proteinuria after 48 weeks of treatment. A meta-analysis of randomised controlled trials showed that the efficacy of conventional ARB therapy with tretinoin hook, tretinoin glycoside, Cordyceps sinensis or Jinshui bao in DKD was satisfactory (Chen J et al., 2022).

### Factors influencing the duration of action of natural products on the AMPK signaling pathway

#### Structural modification of natural products

Resveratrol is a polyphenolic natural product. The phenolic hydroxyl group and other functional groups in its chemical structure give it a certain biological activity, but also affect its stability. *In vivo*, resveratrol may be metabolically transformed, and its activation of the AMPK signaling pathway is relatively short-lived. Its free form in circulating plasma is rare, and bioavailability is less than 1% for 28 days, even with repeated dosing of up to 5 g/day. This results in a limited sustained activation of the AMPK signaling pathway. However, its stability can be improved by structural modifications such as nano-formulations or complexes with other molecules. In a randomised, double-blind, placebo-controlled pharmacokinetic study in healthy subjects, resveratrol in a water-soluble micelle/hydrogel composite in powder form (RF-20) provided higher free resveratrol bioavailability when compared to unformulated resveratrol with 98% purity and pharmacokinetic properties. The enhancement in bioavailability was more when supplemented in sachet (12.98-fold) form than the capsule (10.48-fold) with improved absorption (*C*_max_ = 50.97 ± 15.82 ng/mL), circulation half-life (*t*_1/2_ = 7.01 ± 1.44 h), and sustained delivery (*T*_max_ = 4.71 ± 0.73 h), as compared to the unformulated form (*C*_max_ = 15.07 ± 5.10 ng/mL; *t*_1/2_ = 1.58 ± 0.65 h; *T*_max_ = 1.21 ± 0.42 h) (Joseph et al., 2022).

#### Delivery methods

When natural products are administered orally, some of them are broken down or metabolised due to digestion and absorption in the gastrointestinal tract, which affects the effective concentration and duration of the natural product in reaching the target cells and activating the AMPK signalling pathway. For example, when Flavopiridol is taken orally, absorption in the gastrointestinal tract is incomplete and only a portion of Flavopiridol is able to enter the blood circulation and reach the cells to take effect. In contrast, berberine sulphate, a widely used injectable veterinary drug, was administered intravenously. The absolute bioavailability of berberine was found to be 0.37% ± 0.11% and may be relatively long lasting (Feng et al., 2021).

## Limitations

### Low bioavailability

The bioavailability of active metabolites in many natural products is often low. These metabolites may not be readily absorbed in the gastrointestinal tract, or they may be rapidly metabolized and broken down after entering the bloodstream. For instance, certain natural polyphenolic metabolites, despite their potent activation of AMPK in in vitro experiments, struggle to cross the intestinal mucosa into the bloodstream when administered orally. This difficulty is largely due to their chemical structures. Additionally, once these metabolites reach the liver, they are quickly metabolized into inactive metabolites, preventing them from achieving effective therapeutic concentrations in the body.

### Unclear mechanism of action

Although natural products are recognized for their impact on the AMPK signaling pathway, potentially mitigating diabetes and its complications, the precise mechanisms of action remain elusive. These metabolites might influence multiple signaling pathways simultaneously, complicating the understanding of their effects. For example, a natural product could affect both the AMPK pathway and other inflammation-related pathways. Distinguishing whether the improvements in diabetes and its complications are due to the action of the AMPK pathway alone or from a synergistic effect of multiple pathways is challenging. Furthermore, it is uncertain whether effects observed at the cellular level or in animal models translate effectively into therapeutic benefits in humans.

### Lack of large-scale clinical trials

Compared to chemically synthesized drugs, natural products used for treating diabetes and its complications have been subject to fewer large-scale clinical trials. This discrepancy largely stems from the complex composition of natural products and challenges associated with standardization. The lack of extensive clinical trials makes it difficult to accurately evaluate their efficacy, safety, and the risks associated with long-term use. While some natural products have demonstrated improvements in blood glucose levels or alleviation of complications in preliminary studies with small sample sizes, verifying their effectiveness and safety in larger populations remains necessary.

## Discussion and conclusion

**FIGURE 3:**
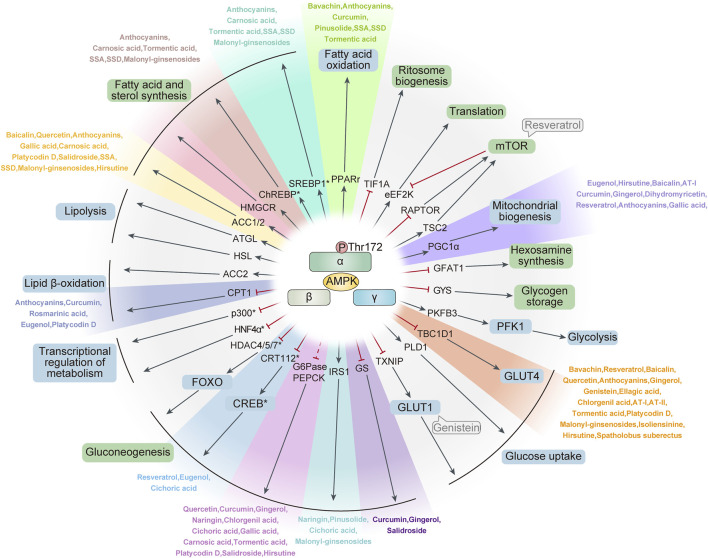
AMPK regulates a variety of metabolic processes. The pathways modulated by AMPK are grouped into four general categories: protein metabolism, lipid metabolism, and glucose metabolism, showcasing the broad range of processes regulated by AMPK. mTOR is modulated by AMPK while simultaneously modulating several direct or indirect targets of AMPK. This is illustrated by arrows from mTOR to its targets, emphasizing the complex relationship between these two signaling pathways. Transcriptional regulators are indicated by an asterisk. It is important to note that only a subset of AMPK substrates is included in the figure. Abbreviations: ChREBP, carbohydrate-responsive element-binding protein; CREB, cAMP response element-binding protein; FOXO1(forkhead box protein O 1), forkhead box protein O; HDAC, histone deacetylase; HMGCR, HMG-CoA reductase; HNF4α, hepatocyte nuclear factor 4α; MFF, mitochondrial fission factor; PGC1α, peroxisome proliferator-activated receptor-γ co-activator 1α; PLD1, phospholipase D1; SREBP1, sterol regulatory element-binding protein 1; TFEB, transcription factor EB. Notations: Black Arrow (↓): Indicates promotion. Red Rough Arrow (⊥): Indicates inhibition.

By targeting the AMPK signaling pathway with natural products for the treatment of diabetes mellitus and its complications, we have found that AMPK, as a key molecule in metabolic regulation, is critical in maintaining energy homeostasis, regulating insulin sensitivity, and reducing inflammation (AL-Ishaq et al., 2019; Gong et al., 2024; Shi et al., 2024). Activation of AMPK can significantly enhance glucose uptake, improve lipid metabolism, and promote autophagy (Wang et al., 2024). Moreover, it alleviates IR and reduces the incidence of diabetes and its complications (Galicia-Garcia et al., 2020). Therefore, the activation of the AMPK signaling pathway has emerged as a potential therapeutic strategy for controlling the progression of T2DM (Figure 3).

This review examines the effects of Phenols, flavonoids, Terpenoids, Alkaloids, Quinones natural products on AMPK activation and their antidiabetic potential. Studies have demonstrated that natural products such as resveratrol and berberine can improve insulin sensitivity by directly or indirectly activating the AMPK signaling pathway, while also reducing IR and inflammation by regulating glucose metabolism and lipolysis (Um et al., 2010; Hardie, 2011; Timmers et al., 2011; Fu et al., 2016; Gasparrini et al., 2016; Francini et al., 2019; Pastor et al., 2019; Den Hartogh et al., 2020; Vlavcheski et al., 2020; Yu et al., 2021; Wang M et al., 2022). Notably, resveratrol and its derivatives exhibit significant effects on improving IR and mitigating inflammation via the AMPK pathway (Um et al., 2010). In a randomised, placebo-controlled trial, resveratrol supplementation played a role in regulating glucose haemostasis, inflammation and oxidative stress in patients with T2DM (Mahjabeen et al., 2022). Additionally, this paper discusses the role of AMPK in combating oxidative stress and regulating autophagy. Activation of AMPK by natural products can inhibit apoptosis, reduce the release of inflammatory factors, and has shown effective hypoglycemic effects in various experimental models. The antioxidant and anti-inflammatory properties of flavonoids, in particular, make them promising therapeutic agents for managing diabetic complications. Although these natural products have shown strong therapeutic potential in experimental models, challenges remain in their clinical application (Chalotra et al., 2024). The specific mechanisms of action for many natural products are not yet fully understood, and their bioavailability, metabolic pathways, and potential toxic side effects require further investigation (Guo et al., 2023). Moreover, due to the structural complexity and diversity of natural products, optimizing the extraction and formulation of efficient and safe AMPK activators from these metabolites remains a focus for future research. Interdisciplinary collaboration will be crucial in advancing the development of natural products targeting AMPK signaling pathways. By combining expertise from chemistry, biology, medicine, and engineering, it is possible to explore the properties and mechanisms of natural products from multiple perspectives. For example, chemical synthesis techniques can be used to modify the structure of natural products to enhance their activity and bioavailability, while bioengineering technologies can help develop efficient extraction and purification methods to ensure the quality and stability of these natural metabolites (Popova and Bankova, 2023). (Figure 3).

Early studies showed that AMPK as a key molecule in metabolic regulation has an important role in maintaining cellular energy homeostasis. These studies have mainly focused on the role of AMPK in the insulin signaling pathway, for example, improving glucose uptake and lipid metabolism. However, many studies have verified the utility of chemically synthesized AMPK agonists, such as drugs such as AICAR and metformin. Although these studies demonstrate the effectiveness of AMPK activation for diabetes treatment, the side effects of its chemical agonist (e.g., hepatotoxicity) limit its clinical use.

In contrast, the current study has increasingly focused on the role of natural products in AMPK activation. For example, natural products such as resveratrol and berberine have been shown to directly or indirectly activate the AMPK pathway, while exhibiting lower toxic side effects and good tolerability. The current study not only verified the regulatory role of natural products on the AMPK signaling pathway, but also explored its role in anti-inflammatory, anti-oxidation and autophagy regulation, demonstrating a broader therapeutic potential.

At the same time, big data and artificial intelligence (AI) technologies can offer new approaches and methods for researching natural products (Singla et al., 2023). By analyzing and mining large volumes of experimental data, potential associations between natural products and the AMPK signaling pathway can be identified, allowing for the prediction of therapeutic effects and potential risks. Moreover, AI algorithms can be employed to optimize the formulation and administration of natural products, thereby improving the precision and effectiveness of treatment.

From a more comprehensive perspective, current studies explore the multiple mechanisms of action of natural products, such as resistance to oxidative stress, regulation of autophagy, and inhibition of the release of inflammatory factors, which is in sharp contrast to previous studies focusing on a single function. In addition, previous studies have focused more on the experimental results of natural products in cells and animal models, while the current work has begun to focus on the systematic study of their bioavailability, metabolic paths and toxic side effects.

In terms of experimental methods, modern research combines big data analysis and artificial intelligence technology to more efficiently explore the potential relationship between natural products and AMPK signaling pathways, and predict their therapeutic effects and risks. This research model based on technological innovation has significantly improved research efficiency and accuracy, while previous research has mainly relied on traditional experimental verification methods and progressed relatively slowly.

In terms of clinical research, multi-center, large-sample clinical trials should be conducted to further verify the safety and efficacy of natural products in the treatment of diabetes and its complications. Simultaneously, a long-term follow-up mechanism should be established to assess the impact of natural products on patient outcomes and long-term efficacy. Additionally, the combined use of natural products with conventional diabetes treatments could be explored to achieve synergistic effects and enhance therapeutic outcomes.

In summary, natural products targeting the AMPK signaling pathway show significant potential in the prevention and treatment of diabetes and its complications. Future research should focus on in-depth studies of the underlying mechanisms, exploration of pharmacological properties, and evaluation of the clinical applications of these natural products, with the goal of providing safer and more effective treatment options for diabetic patients.

## Glossary

DM: Diabetes mellitus
TIDM: Type 1 diabetes mellitus
T2DM: Type 2 diabetes mellitus
AMPK: Adenylate-activated protein kinase
IR: Insulin resistance
TNF-α: Tumor necrosis factor-α
IL-6: interleukin-6
BMI: Body mass index
CaMKKβ: Calcium/calmodulin-dependent kinase kinase β
LKB1: Liver kinase B1
PP: Protein phosphatases
T172: The phosphorylation of threonine 172
ATP: Adenosine Triphosphate
AMP: Adenosine monophosphate
ADP: Adenosine diphosphate
mTOR: mammalian target of rapamycin
NAFLD: Non-alcoholic fatty liver disease
Aβ: Amyloid beta
NLRP3: NOD-like receptor thermal protein domain associated protein 3
ULK1: Unc-51-like kinase 1
ACC: Acetil Coenzyme A Carboxylase
CPT1: Carnitine Palmitoyltransferase 1
GBD: Glycogen-Binding Domain
GLUT 4: Glucose Transporters 4
HMG-CoA reductase: 3-hydroxy-3-methyl-glutaryl-coenzyme A reductase
PFK-2: 6-Phosphofructo-2-Kinase 2
SREBP-1c: Sterol Response Element Binding Protein-1c
EGCG: epigallocatechin-3-gallate epigallocatechin-3-gallate
TORC: Target of Rapamycin Complex
GSK3β: glycogen synthase kinase 3β
G6Pase: Glucose-6-phosphatase
C/EBPβ: CCAAT/enhancer binding protein β
FOXO1: Forkhead box protein O 1
PPAR: Peroxisome proliferator-activated receptor
GOT: Glutamic oxaloacetic transaminase
GPT: Glutamic pyruvic transaminase
FAS: Fatty acid synthase
ACC: Acetyl-CoA carboxylase
CPT1: Carnitine Palmitoyltransferase One
HFD: High-fat diet
CRTC2: CREB Regulated Transcription Coactivator-2
CREB: cAMP Response Element-Binding Protein
SIRT: Silent Mating-Type Information Regulation 2 Homolog
PGC-1α: PPAR Coactivator-1α
Akt: Protein Kinase B
FFA: Free fatty acid
IRS: insulin receptor
PI3K: Phosphatidylinositol 3-kinase
SIRT1: Sirtuin1
TFAM: Mitochondrial transcription factor A
NRF-1: Nuclear respiratory factor-1
ER: Endoplasmic reticulum
PEPCK: Phosphoenolpyruvate Carboxykinase
Apo C-III: Apolipoprotein C-III
Thr172: Threonine172
STZ: Streptozotocin
FBG: fasting blood glucose
TC: Total cholesterol
TG: Triglycerides
HDL: high-density lipoprotein
LDL-C: Low-density lipoprotein cholesterol
AICAR: 5-aminoimidazole-4-carboxamide ribonucleoside
DCM: Diabetic cardiomyopathy
FABP4: Fatty acid binding protein
LPL: lipoprotein lipase
ERK1/2: Extracellular-regulated kinase 1/2 (ERK1/2)
JNK: c-Jun-N-terminal kinase
MAPK: Mitogen-activated protein kinase
AST: Aspartate aminotransferase
ALT: Alanine aminotransferase
PERK: RNA-activated protein kinase-like ER resident kinase
NF-κB: Nuclear factor kappa B.
