# Corrigendum: Natural products targeting AMPK signaling pathway therapy, diabetes mellitus and its complications

**DOI:** 10.3389/fphar.2025.1604573

**Published:** 2025-04-14

**Authors:** Min Li, Lu Ding, Liyuan Cao, Zepeng Zhang, Xueyan Li, Zirui Li, Qinjing Xia, Kai Yin, Siyu Song, Zihan Wang, Haijian Du, Daqing Zhao, Xiangyan Li, Zeyu Wang

**Affiliations:** ^1^ Northeast Asia Research Institute of Traditional Chinese Medicine, Key Laboratory of Active Substances and Biological Mechanisms of Ginseng Efcacy, Ministry of Education, Jilin Provincial Key Laboratory of Bio-Macromolecules of Chinese Medicine, Changchun University of Chinese Medicine, Jilin, China; ^2^ Research Center of Traditional Chinese Medicine, The Affiliated Hospital to Changchun University of Chinese Medicine, Jilin, China

**Keywords:** AMPK, natural products, type 2 diabetes, complications, mechanism

In the published article, an **Author name** was incorrectly written as [Liyan Cao]. The correct spelling is [Liyuan Cao].

The authors apologize for this error and state that this does not change the scientific conclusions of the article in any way. The original article has been updated.

